# Failure of endocytic flux in Donnai-Barrow syndrome caused by LRP2 p.C1400R

**DOI:** 10.1172/jci.insight.199341

**Published:** 2026-04-23

**Authors:** Andrew Beenken, Tian H. Shen, Aryan Ghotra, Hediye Erdjument-Bromage, Jeong Lee, Jared S. Kushner, Rachel E. Sturley, Atlas Khan, Jeffrey R. Arace, Leora Kronenberg, Lucy D. Shen, Gabriel H. Rahmani, Patricia K. Donahoe, Thomas A. Neubert, Frances A. High, Ora A. Weisz, Jonathan Barasch

**Affiliations:** 1 Division of Nephrology, Columbia University Vagelos College of Physicians and Surgeons, New York, New York, USA.; 2 Department of Neuroscience, NYU Grossman School of Medicine, New York, New York, USA.; 3 Division of Cardiology, Columbia University Vagelos College of Physicians and Surgeons, New York, New York, USA.; 4 Pediatric Surgical Research Laboratories and Department of Surgery, Massachusetts General Hospital and Harvard Medical School, Boston, Massachusetts, USA.; 5 Department of Pediatrics, Massachusetts General Hospital, Boston, Massachusetts, USA.; 6 Renal-Electrolyte Division, University of Pittsburgh School of Medicine, Pittsburgh, Pennsylvania, USA.

**Keywords:** Cell biology, Nephrology, Genetic diseases, Protein traffic, Proteomics

## Abstract

Donnai-Barrow syndrome (DBS) arises from loss-of-function (LoF) variants in the endocytic receptor low-density lipoprotein receptor–related protein 2 (LRP2; or megalin) and is characterized by low–molecular weight proteinuria and developmental abnormalities. Urinary proteomics of 9 patients with DBS revealed that the urinary proteome of a DBS patient with the missense variant LRP2 p.C1400R was indistinguishable from that of patients with splice site, nonsense, or frameshift mutations. A CRISPR mouse model of the variant was generated to determine the mechanism of LoF and proteinuria. The mutant LRP2 was expressed and observed to dimerize and localize to the proximal tubule apical membrane. However, both fluid-phase and receptor-mediated endocytosis was impaired in the context of a general perturbation of endocytic flux. Immunofluorescence revealed aberrant endocytic recycling with mislocalized RAB11^+^ and TFR1^+^ compartments and enlarged lysosomes. Structural modeling showed that the LRP2 assembly likely tolerates the cysteine-to-arginine substitution at the cell surface, but at endosomal pH the variant introduced steric clashes that may disrupt intramolecular interfaces and disturb receptor recycling. These findings point to the importance of LRP2 recycling for global endocytic flux and offer a blueprint for leveraging patient-specific alleles to dissect proximal tubule function.

## Abstract 2

A CRISPR mouse model of a patient-derived rare disease variant provides a handle for understanding receptor biology in the kidney

## Introduction

Low-density lipoprotein receptor–related protein 2 (LRP2; or megalin) is a 600 kDa multiligand endocytic receptor abundantly expressed at the apical surface of the proximal tubule (PT), where it mediates reabsorption of low–molecular weight (LMW) proteins from the glomerular filtrate (1–10) as well as albumin reabsorption and transcytosis (11–14). LRP2 interacts with its coreceptor cubilin at the apical membrane to bind a diverse repertoire of ligands (13, 15–18), preventing their loss into the urine. Among the many LMW proteins undergoing LRP2-mediated endocytosis in the PT are vitamin-binding proteins (19, 20), protease–protease inhibitor complexes (21, 22), lipoproteins (22–26), and lipocalins (27, 28). Outside the kidney, LRP2 is expressed in diverse epithelia, including that of the thyroid, parathyroid, inner ear, alveoli, eye, and choroid plexus (29–36), and LRP2 plays key roles during the development of the central nervous system (CNS) (37–42). Loss-of-function (LoF) variants in *LRP2* lead to Donnai-Barrow syndrome (DBS; OMIM 222448), a rare autosomal recessive disorder characterized by proteinuria and glomerulotubular defects as well as a constellation of CNS developmental abnormalities including craniofacial dysmorphism, ocular abnormalities, sensorineural hearing loss, agenesis of the corpus callosum, and structural birth defects (16, 43–54). To date, most reported *LRP2* variants leading to DBS are thought to result in a loss of expression of LRP2 (43, 50), and functional studies of DBS in mice have been conducted in *Lrp2*-knockout models as opposed to knockin models of DBS variants (44) where domain-specific physiologic mechanisms can be explored.

Recently, an in vitro model of the DBS-causing variant LRP2 p.R3192Q revealed expression of this LoF variant in patient-derived inducible pluripotent stem cells that had been differentiated to neuroepithelial and kidney cell types (55). In the in vitro model, ligand-induced decay of LRP2 was proposed as the mechanism of LoF. LRP2 p.R3192Q was observed to bind sonic hedgehog (Shh) in neuroepithelial cells but failed to recycle, and the ligand-receptor complexes were trafficked to lysosomes for degradation. DBS-causing variants of *LRP2* thus may induce PT dysfunction through diverse mechanisms (55), including abnormal intracellular trafficking.

LRP2 captures and releases ligand during its fast recycling between apical membrane and endosomes (5, 56–59), and its presence in the PT is essential for normal patterns of endocytic flux (60), yet the structure-function relationships underlying LRP2 physiology are only beginning to be elucidated (61, 62). Studies of LRP2 LoF in mouse models based on patient-derived missense variants provide an opportunity for identifying structure-function relationships critical for endocytosis pathways in the PT. If patient-derived LRP2 missense variants express in the PT apical membrane yet nonetheless exhibit LoF, those missense variants could serve as probes of LRP2 function and apical endocytic trafficking in the PT. LRP2 is a member of the broader LRP subfamily, with which it shares both a highly conserved architecture (10, 63–66) and a similar repertoire of cognate ligands (18, 22, 67), so discoveries of structure-function relationships in LRP2 will be of consequence for the LRP subfamily as a whole (68).

Expanding the functional understanding of LRP2 is critical for future translational research to prevent kidney injury, since LRP2 is the gateway in the PT for toxins causing acute kidney injury such as polymyxin (69–72), gentamicin (72, 73), myoglobin (74), and light chains (75). Functional studies of LRP2 will also inform the study of chronic kidney disease, since GWAS studies have linked LRP2 to estimated glomerular filtration rate and urate levels (76–83), and LRP2-mediated protein uptake contributes to tubular injury in models of nephrotic syndrome (84). In a study of a cohort of 6 families carrying homozygous pathogenic DBS variants, in addition to LMW proteinuria, all patients demonstrated elevated biomarkers of tubular injury (44).

In the present study, the pathogenic consequences of the DBS missense variant LRP2 p.C1400R are explored in a CRISPR/Cas9 knockin mouse model harboring the orthologous mutation, *Lrp2* p.C1401R. This investigation provides a template for the study of PT function using a patient-derived LRP2 variant as a mechanistic probe.

## Results

### The DBS urinary proteome reveals a shared pattern of LMW proteinuria across variants.

**Figure 1:**
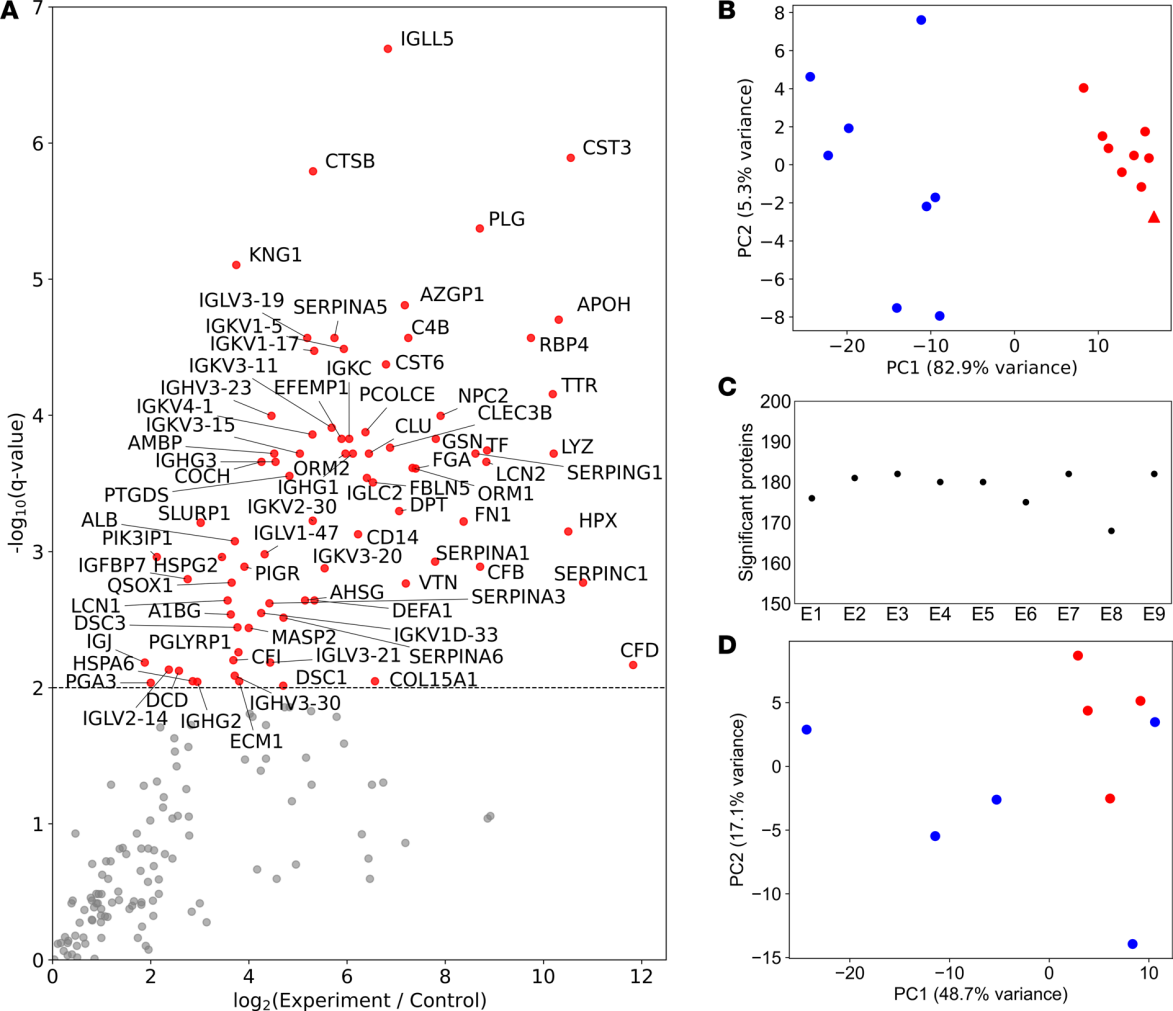
Donnai-Barrow syndrome has a consistent proteomic signature. (**A**) Log_2_(experiment/control) of intensities is plotted against –log_10_(*q* value) for proteins detected in the urine of 9 children with Donnai-Barrow syndrome (DBS) compared with 8 parental controls with *P* values from Welch’s *t* test adjusted with Benjamini-Hochberg procedure (BH-FDR; *q* < 0.01). The 81 significant proteins in this analysis are in red. All significant changes were increases in intensity for proteins in DBS patients relative to controls. (**B**) Principal component analysis (PCA) for the proteins with intensity data for all patients (red, *n* = 9) and controls (blue, *n* = 8). LRP2 p.C1400R is indicated as a triangle. (**C**) Total significant proteins (BH-FDR *q* < 0.01) identified by either Welch’s *t* test or Fisher’s exact test in each experimental sample. *LRP2* p.C1400R is sample E5. (**D**) PCA for the proteins seen in all patients, with females (red, *n* = 4) and males (blue, *n* = 5) showing no significant sex effect across PC1 or PC2 by Welch’s *t* test.

To define the urinary proteome associated with DBS, liquid chromatography/tandem mass spectrometry (LC-MS/MS) analysis was performed on urine samples from 9 children with DBS and 8 healthy parental controls (Table 1 and Supplemental Figure 1; supplemental material available online with this article; https://doi.org/10.1172/jci.insight.199341DS1). Of the 9 children, 8 harbored frameshift, splice site, or nonsense mutations, while 1 child had a missense variant, LRP2 p.C1400R, detected in only a single allele. This patient is presumed to be a compound heterozygote with the second allele remaining unidentified by exome sequencing. Thus, an additional mutation may contribute to the phenotype in this patient. All had clinically confirmed DBS (Frances A. High and Patricia K. Donahoe, unpublished observations). Statistical comparison between the DBS and control groups revealed 81 proteins with significantly altered abundance by Welch’s 2-tailed *t* test with Benjamini-Hochberg correction (BH-FDR *q* < 0.01) conducted on log_2_-transformed intensity data (Figure 1A), with an additional 105 proteins identified as significant by Fisher’s exact test for proteins with intensity data too sparse for *t* test analysis (Supplemental Figure 2). Many of the proteins identified in DBS urine are classical LRP2 ligands, including retinol-binding protein 4 (RBP4), lipoprotein lipase (LPL), angiotensinogen (AGT), β_2_-microglobulin (B2M), lipocalin-2 (LCN2), antithrombin III (SERPINC1), and α_1_-microglobulin (AMBP) (18). These results are consistent with published reports of the urinary proteome of megalin-knockdown mouse models (85).

Within the patient cohort, 2 patients had elevated serum creatinine to 1.7 (Table 1), potentially suggesting a glomerular injury or more likely a tubulointerstitial injury from LRP2 LoF in these patients, consistent with the findings in other DBS cohorts (44). While the filtrate normally contains fragments of different proteins (86), the increased presence of tryptic peptides of fibronectin (FN1) as well as COL15A1 in all DBS urines compared with controls may represent a signal for tubulointerstitial fibrotic injury in these patients (Figure 1A) (87–89).

Unsupervised multivariate analysis was conducted on the proteins that had quantifiable intensities across all control and experimental samples, demonstrating a clear separation between the DBS patient group and the control group in a principal component analysis (PCA) (Figure 1B). The urinary proteome of the patient with LRP2 p.C1400R clustered with that of the other DBS variants, indicating a consistent composition of the DBS urinary proteome. This finding was further emphasized by a similar number of significant proteins identified across all experimental samples (Figure 1C). The clustering of the LRP2 p.C1400R variant patient (E5) within the DBS group suggests that this specific missense mutation phenocopies the broader urinary proteomic consequences of LRP2 frameshift, nonsense, and splice site mutations causing DBS (Figure 1, B and C). A sex-stratified PCA of proteins quantifiable in all DBS patients did not reveal systematic sex-based differences in the urinary proteomes of male and female DBS patients (Figure 1D). The similar degree of LMW proteinuria observed across the DBS patient cohort regardless of variant type raised the possibility that distinct molecular defects converge on a common functional failure.

### LRP2 p.C1401R localizes to the apical membrane in a CRISPR mouse model.

To dissect the pathophysiologic basis of the proteinuric phenotype in the missense variant LRP2 p.C1400R and to test the hypothesis that DBS can result from diverse molecular defects, a knockin CRISPR mouse model of the orthologous murine variant, *Lrp2* p.C1401R, was generated (Supplemental Figure 3, A–C).

**Figure 2:**
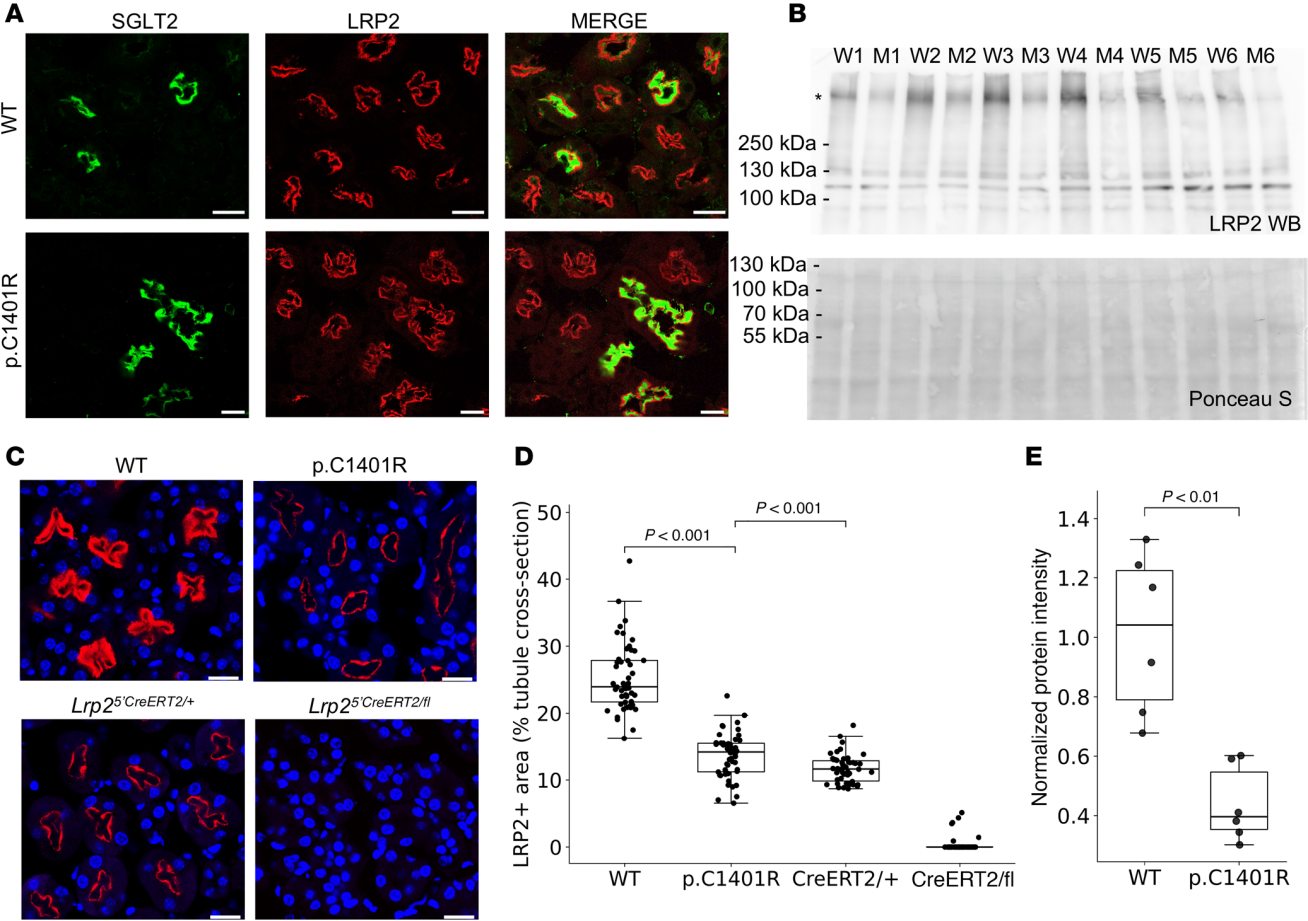
LRP2 p.C1401R is expressed apically. (**A**) Immunofluorescence (IF) for SGLT2 (green) and LRP2 (red) in kidney cryosections from WT *Lrp2* (WT) and *Lrp2* p.C1401R mice (p.C1401R). The mutant protein shows apical localization in proximal tubules, similarly to WT, and localizes adjacent to SGLT2. Scale bars: 20 μm. Representative images from *n* = 3 mice. (**B**) An LRP2 Western blot (WB) of 6 WT post-nuclear supernatant (PNS) samples (W1–W6) and 6 *Lrp2* p.C1401R PNS samples (M1–M6), with associated Ponceau S total protein loading control. The asterisk marks the LRP2 band. Representative of *n* = 3 technical replicates. (**C**) LRP2 apical expression is assessed with fluorophore-conjugated anti-LRP2 (red). Scale bars: 20 μm. Representative images from *n* = 3 mice. (**D**) Quantitation of LRP2 staining of tubule cross sections from **C** by Welch’s *t* test. *N* = 50 tubule cross sections from *n* = 3 mice from each genotype: WT, p.C1401R, *Lrp2*^5′CreERT2/+^ (CreERT2/+) as a haploinsufficient control, and *Lrp2*^5′CreERT2/fl^ (CreERT2/f) as a knockout control. Pairwise comparisons are shown for WT versus p.C1401R and p.C1401R versus CreERT2/+ to test the hypothesis that p.C1401R reduces LRP2 expression. (**E**) Quantitation of LRP2 WB from **B**, by Welch’s *t* test; *n* = 6 WT *Lrp2* and *n* = 6 *Lrp2* p.C1401R mice. Box plots display the median (center line) and interquartile range (box boundaries), with whiskers extending to the most extreme data point within 1.5 times the interquartile range; individual data points are overlaid.

In both WT and knockin *Lrp2* p.C1401R mice, LRP2 and the S1/S2 segment proximal tubule apical brush border marker sodium-glucose cotransporter 2 (SGLT2) (90, 91) preserved their relative localizations, with LRP2 immediately subapical to SGLT2 on immunofluorescence (IF) (Figure 2A). There was no apparent evidence on IF of LRP2 being retained in the endoplasmic reticulum. Quantitative IF using Alexa Fluor 594–conjugated anti-LRP2 showed that LRP2^+^ fractional area was reduced by 46% in *Lrp2* p.C1401R mice compared with WT (WT, 25.2% ± 5.1%; *Lrp2* p.C1401R, 13.63% ± 3.3%; *P* = 6.13 × 10^–23^ by Welch’s *t* test; Figure 2, C and D) and was slightly increased relative to the LRP2-haploinsufficient control (*Lrp2*^5′CreERT2/+^, 11.68% ± 2.1%; *P* = 6.64 × 10^–4^ by Welch’s *t* test). The conditional-knockout negative control had negligible LRP2 expression (*Lrp2*^5′CreERT2/fl^, 0.38% ± 1.2%) (Figure 2, C and D).

Overall LRP2 expression levels in WT and *Lrp2* p.C1401R mice were also compared in the post-nuclear supernatant with quantitative Western blot normalized with Ponceau S staining of total protein (92, 93). In a representative technical replicate, normalized LRP2 WT intensity was 1.014 ± 0.272 compared with 0.438 ± 0.128 for LRP2 p.C1401R, a 57% reduction in protein expression in the mutant (*P* = 0.002 by Welch’s 2-sample *t* test) (Figure 2, B and E). This finding was reproducible across 3 technical replicates, with a linear mixed model analysis yielding an estimated marginal mean for protein intensity in WT of 31,092 ± 2,818 AU compared with 15,306 ± 2,823 AU in LRP2 p.C1401R, significant using Satterthwaite’s method (*P* = 0.0007) (94). These results indicate that despite its apical localization, the *Lrp2* p.C1401R mouse model exhibits a decrease in steady-state LRP2 protein expression levels, due to either reduced protein stability or failure of protein recycling, leading to degradation.

### LRP2 p.C1401R retains the dimeric assembly of WT LRP2 following biosynthesis.

**Figure 3:**
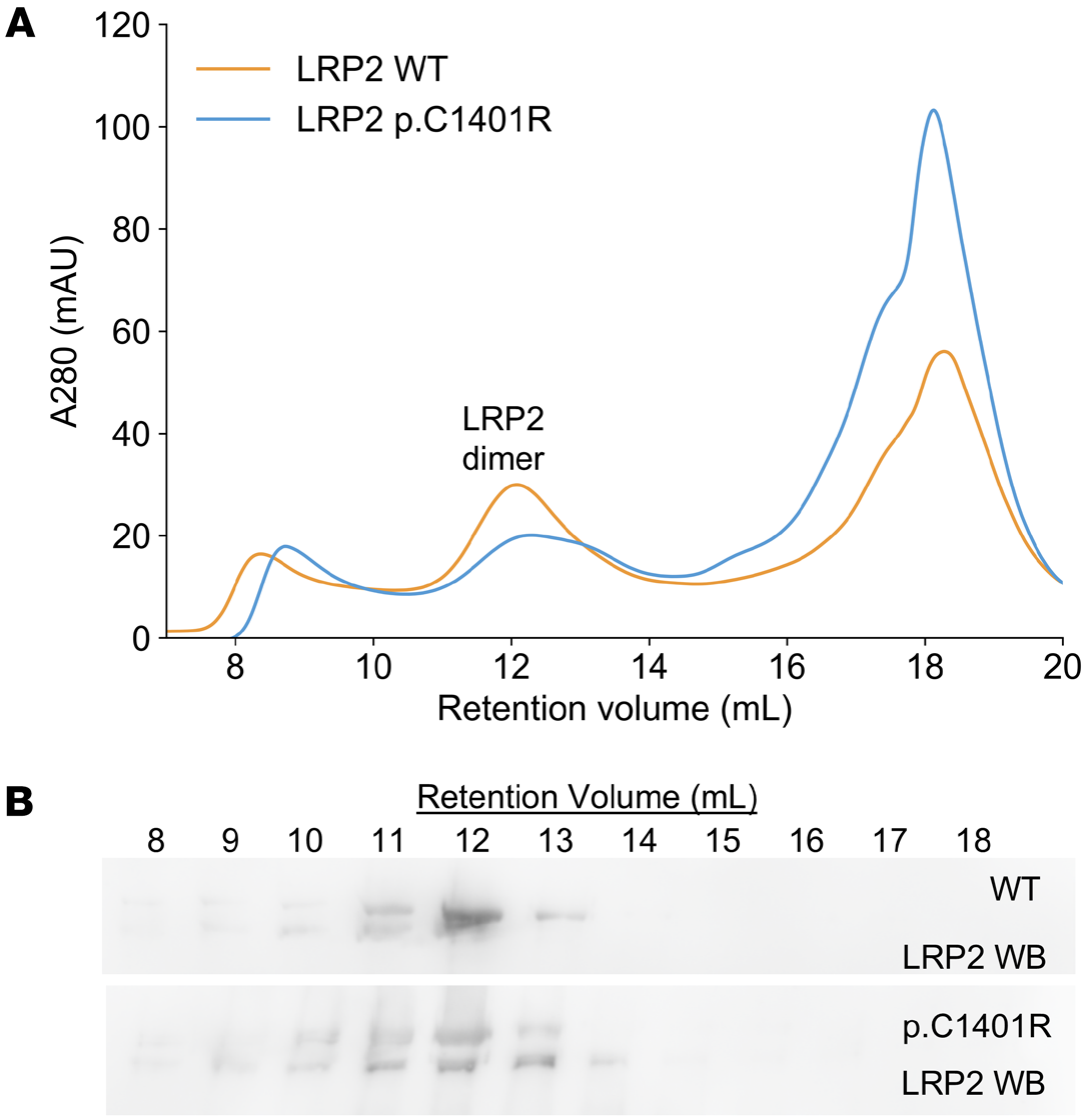
LRP2 p.C1401R folds as a dimer. (**A**) Size exclusion chromatography (SEC) analysis of LRP2 WT (orange) and LRP2 p.C1401R (blue). The chromatographic peak containing LRP2 is labeled. Representative of *n* = 3 experiments. (**B**) LRP2 Western blot (WB) of SEC fractions aligned with the chromatogram in **A** showing similar elution patterns for WT and LRP2 p.C1401R by anti-LRP2 staining.

Endogenous LRP2 p.C1401R was purified to determine whether it retained the dimeric assembly observed in WT LRP2 or whether it failed to adopt the native WT conformation, thus predisposing it to degradation and reducing apical expression. Endogenous LRP2 p.C1401R was isolated from homozygous mouse kidneys using anion exchange and size exclusion chromatography (SEC) in an analogous protocol to that used for WT LRP2 (61). The SEC chromatogram of LRP2-containing anion exchange fractions demonstrated a peak at a retention volume of 12 mL for LRP2 p.C1401R (Figure 3A, blue tracing) matching that of the dimer peak of the 1.2 MDa LRP2 WT protein (Figure 3A, orange tracing). Western blotting confirmed that LRP2 p.C1401R eluted from SEC similarly to WT (Figure 3B), suggesting that LRP2 p.C1401R retains the dimeric assembly observed in WT LRP2. These results demonstrate that a population of LRP2 p.C1401R navigates the endoplasmic reticulum and Golgi apparatus and undergoes the expected dimerization during protein folding. Thus, the primary pathophysiologic defect in the LRP2 p.C1401R model likely does not lie in LRP2 biosynthesis.

To confirm the presence of LRP2 p.C1401R in the protein sample and exclude the possibility that WT LRP2 may be confounding the biochemical analysis, tryptic peptides from an LRP2 p.C1401R preparation were characterized by LC-MS/MS. In WT LRP2, tryptic digestion generates a peptide spanning the region around amino acid 1401 with the sequence TCEDVNECDIPGF^1401^CSQHCVNMR (calculated monoisotopic mass, MH^+1^ = 2,728.07 Da, MH^2+^ = 1,364.54 Da). In LRP2 p.C1401R there is instead the tryptic peptide TCEDVNECDIPGF^1401^R (calculated monoisotopic mass, MH^+1^ = 1,711.70 Da, MH^2+^ = 856.36 Da). Layouts containing predicted experimental masses were created, and total ion current was manually extracted for MS1 peptides at the predicted, doubly charged monoisotopic masses of the LRP2 p.C1401R (Supplemental Figure 4A) and WT LRP2 (Supplemental Figure 4B) peptide spectra. At 52.69 minutes, a clear extracted ion was seen for *z* = 2 at *m*/*z* 856.36 corresponding to the LRP2 p.C1401R peptide spectra (Supplemental Figure 4A), but there was no evidence for the WT LRP2 peptide (*m*/*z* 1,364.54, *z* = 2) (Supplemental Figure 4B). MS2 fragmentation patterns were used to unequivocally demonstrate the presence of the LRP2 p.C1401R peptide. The predicted *y* ion series (C-terminal directed) and *b* ion series (N-terminal directed) for the fragmented LRP2 p.C1401R peptide were observed in the data-independent acquisition (DIA) analysis of MS2 spectra in the wide window spanning *m*/*z* 856.36 at approximately 52 minutes (Supplemental Figure 4C). Thus, the biochemical behavior of the homozygous LRP2 p.C1401R sample reflects a homogenous protein sample composed entirely of mutant LRP2 with no confounding contamination from WT LRP2.

### Lrp2 p.C1401R has evidence of tubular injury despite preserved renal function.

Given the gross competency of LRP2 p.C1401R to form a dimeric structure and target the apical membrane, its reduced expression may be associated with broader cellular defects. Since reduced LRP2 expression has been associated with tubular injury (44), we investigated whether signs of injury were evident from histology or injury biomarkers. H&E and periodic acid–Schiff staining showed no gross morphologic abnormalities in *Lrp2* p.C1401R kidneys compared with WT (Supplemental Figure 5, A and B). Further evaluation of LRP2 localization with equilibrium ultracentrifugation showed that LRP2, CUBN, and SGLT2 from WT and *Lrp2* p.C1401R post-nuclear supernatant exhibited similar fractionation profiles (Supplemental Figure 5C). Renal function was preserved at 4 months of age, with no significant difference in blood urea nitrogen (WT, 21.3 ± 3.4 mg/dL; *Lrp2* p.C1401R, 16.0 ± 2.4 mg/dL; not significant by Welch’s *t* test) (Supplemental Figure 5D) and creatinine below the level of detection, <0.2 mg/dL, in all samples. However, tubular injury markers were increased in mutants, with a 20.4-fold elevation in urinary KIM-1/creatinine ratio by ELISA (WT, 1.3 ± 0.6 ng/mg; *Lrp2* p.C1401R, 26.5 ± 9.7 ng/mg; *P* = 0.013 by Welch’s *t* test) and qualitatively increased *Havcr1* (KIM-1) signal by RNAscope (Supplemental Figure 5, E and F), consistent with prior reports of elevated urinary KIM-1 in DBS patients (44). The expression of KIM-1 in the setting of preserved serum creatinine levels is due to the latter’s insensitivity to subclinical tubular injury.

### Receptor-mediated and fluid-phase endocytosis are impaired in Lrp2 p.C1401R mice.

**Figure 4:**
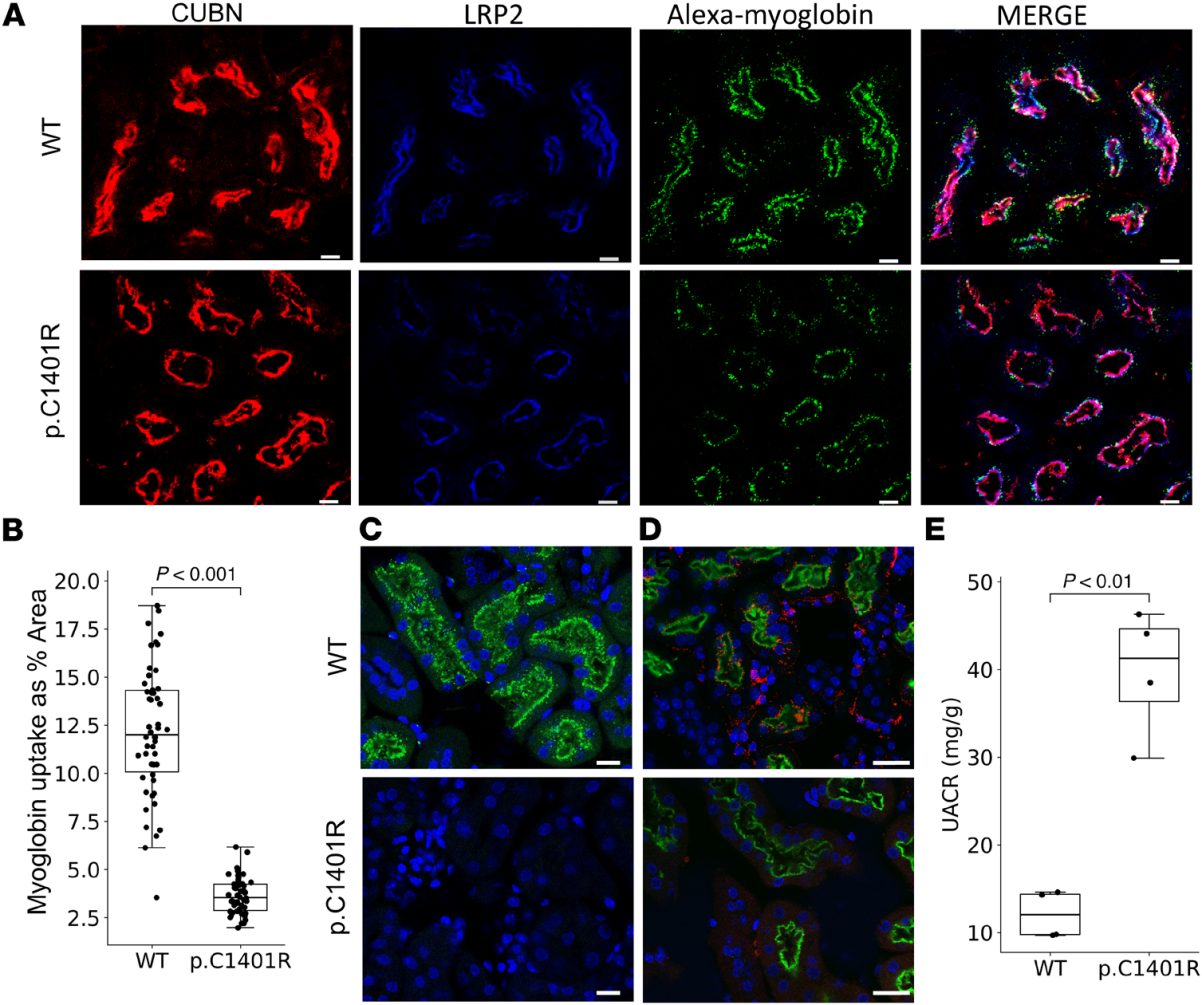
Impairment of receptor-mediated and fluid-phase endocytosis in *Lrp2* p.C1401R. (**A**) Uptake of Alexa Fluor 568–labeled myoglobin (green) following intraperitoneal (i.p.) injection, and IF for cubilin (CUBN, red) and LRP2 (blue), in kidney cryosections from WT *Lrp2* and *Lrp2* p.C1401R mice (p.C1401R). Myoglobin uptake is attenuated in *Lrp2* p.C1401R mice. Scale bars: 10 μm. Representative images from *n* = 3 mice. (**B**) Quantitation of myoglobin uptake by area from *N* = 50 tubular cross sections from *n* = 3 mice using Welch’s *t* test. (**C**) Uptake of FITC-labeled dextran (green) following i.p. injection, with DAPI in blue. Scale bars: 10 μm. Representative images from *n* = 3 mice. (**D**) Uptake of Alexa Fluor 594–labeled albumin (red) following i.p. injection, with DAPI in blue and LRP2 in green. Scale bars: 10 μm. Representative images from *n* = 3 mice. (**E**) Quantitation of urinary albumin/creatinine ratio (UACR) in WT (*n* = 4) and p.C1401R mice (*n* = 4) using Welch’s *t* test. Box plots display the median (center line) and interquartile range (box boundaries), with whiskers extending to the most extreme data point within 1.5 times the interquartile range; individual data points are overlaid.

Given reduced apical expression of LRP2 in the setting of tubular injury, we assessed the functional competence of the PT in *Lrp2* p.C1401R mice. Receptor-mediated endocytosis was directly investigated by detection of uptake following intraperitoneal injection of Alexa Fluor 568–labeled myoglobin, a known LRP2 ligand (74). In *Lrp2* p.C1401R mice, Alexa Fluor 568–myoglobin uptake was attenuated in comparison with WT controls (Figure 4A, representative data, *n* = 3 mice per group). Quantitation by cortical kidney area showed that Alexa-myoglobin–labeled endosomal vesicles constituted 12.2% ± 3.4% of PT area in WT compared with 3.6% ± 0.9% in *Lrp2* p.C1401R mice (*P* = 3.1 × 10^–24^) (Figure 4B).

Fluid-phase endocytosis was next evaluated following intraperitoneal administration of FITC-dextran. *Lrp2* p.C1401R mice showed negligible uptake of FITC-dextran in PT cells compared with WT, pointing to an impairment in fluid-phase endocytosis and a global disturbance in endocytic flux (Figure 4C, representative data, *n* = 3 mice per group). The general dampening of endocytic flux in the context of *Lrp2* p.C1401R is similar to what has been seen in the setting of *Lrp2* knockout (60). Uptake of Alexa Fluor 594–conjugated albumin was also negligible in *Lrp2* p.C1401R compared with WT (Figure 4D, representative data, *n* = 3 mice per group), and urinary albumin/creatinine ratio was elevated in *Lrp2* p.C1401R mice relative to controls (12.1 ± 2.7 mg/g in WT, 39.7 ± 7.3 mg/g in *Lrp2* p.C1401R; *P* = 0.002 by Welch’s *t* test) (Figure 4E). The *Lrp2* p.C1401R mouse model exhibited proteinuria (Supplemental Figure 6A), with qualitative increases in Coomassie blue staining of LMW proteins in the 25 to 75 kDa range in the mutant across urine samples from 4 separate mice. In contrast, proteinuria in heterozygotes was not qualitatively increased in this range (Supplemental Figure 6A). These findings suggest that the human DBS patient most likely harbors a second deleterious LRP2 allele that was not detected by exome sequencing. Sample loading was controlled with urinary creatinine measurements. Quantitative Western blot showed significant increases in normalized intensity in *Lrp2* p.C1401R for retinol-binding protein 4 (RBP4) (WT, 0.005 ± 0.01; *Lrp2* p.C1401R, 0.13 ± 0.08; *P* = 0.045 by Welch’s *t* test; Supplemental Figure 6B) and lysozyme (LYZ) (WT, –0.02 ± 0.0006; *Lrp2* p.C1401R, 0.05 ± 0.03; *P* = 0.022 by Welch’s *t* test; Supplemental Figure 6C).

Given the significant difference in receptor-mediated endocytosis between WT and mutant, CUBN localization was interrogated for apical expression in each genotype using colocalization with the S1/S2 marker SGLT2 (90, 91) and the S1/S2/S3 marker *Lotus tetragonolobus* lectin (LTL) (95). CUBN had apical colocalization with SGLT2 throughout all examined tissue sections (Supplemental Figure 7, A and B, representative images from *n* = 3 mice). CUBN also exhibited apical colocalization with LTL in SGLT2^+^ tubules, but in LTL^+^SGLT2^–^ S3 segment tubules, overall CUBN expression was qualitatively reduced in both WT and mutant, limiting evaluation of apical localization. Reduced CUBN expression in S3 relative to S1/S2 has been previously documented (96). The retained apical localization of CUBN in S1/S2 suggests that perturbed CUBN trafficking is unlikely to underlie the phenotype of the *Lrp2* p.C1401R model.

### LRP2 p.C1401R and WT are differentially biotinylated by V5-APEX2-RAP.

The reduction in receptor-mediated endocytosis suggested the possibility of an impairment in ligand-binding in *Lrp2* p.C1401R. Since the scarce quantity of purified endogenous LRP2 p.C1401R precluded quantitative in vitro binding studies, an experimental protocol was developed to test LRP2 p.C1401R ligand binding ex vivo in explanted mouse kidneys perfused with the LRP2 ligand receptor-associated protein (RAP; or LRPAP1) (22, 97) conjugated to V5-tagged ascorbate peroxidase 2 (V5-APEX2) (Supplemental Figure 8, A and B). The V5-APEX2-RAP fusion protein would enable proximity biotinylation of RAP-bound LRP2 p.C1401R at the luminal surface of the PT and provide direct evidence for a retained capacity for ligand binding in *Lrp2* p.C1401R. The V5-APEX2-RAP fusion construct was perfused for 25 minutes along with biotin-phenol ex vivo into WT and *Lrp2* p.C1401R kidneys followed immediately by a 5-minute pulse of hydrogen peroxide to activate APEX2-mediated proximity biotinylation of proteins situated near bound RAP at the apical surface. This duration of labeling identified primarily apical and early endosomal proteins and largely excluded proteins from the late and recycling endosomal compartments (Table 2).

**Figure 5:**
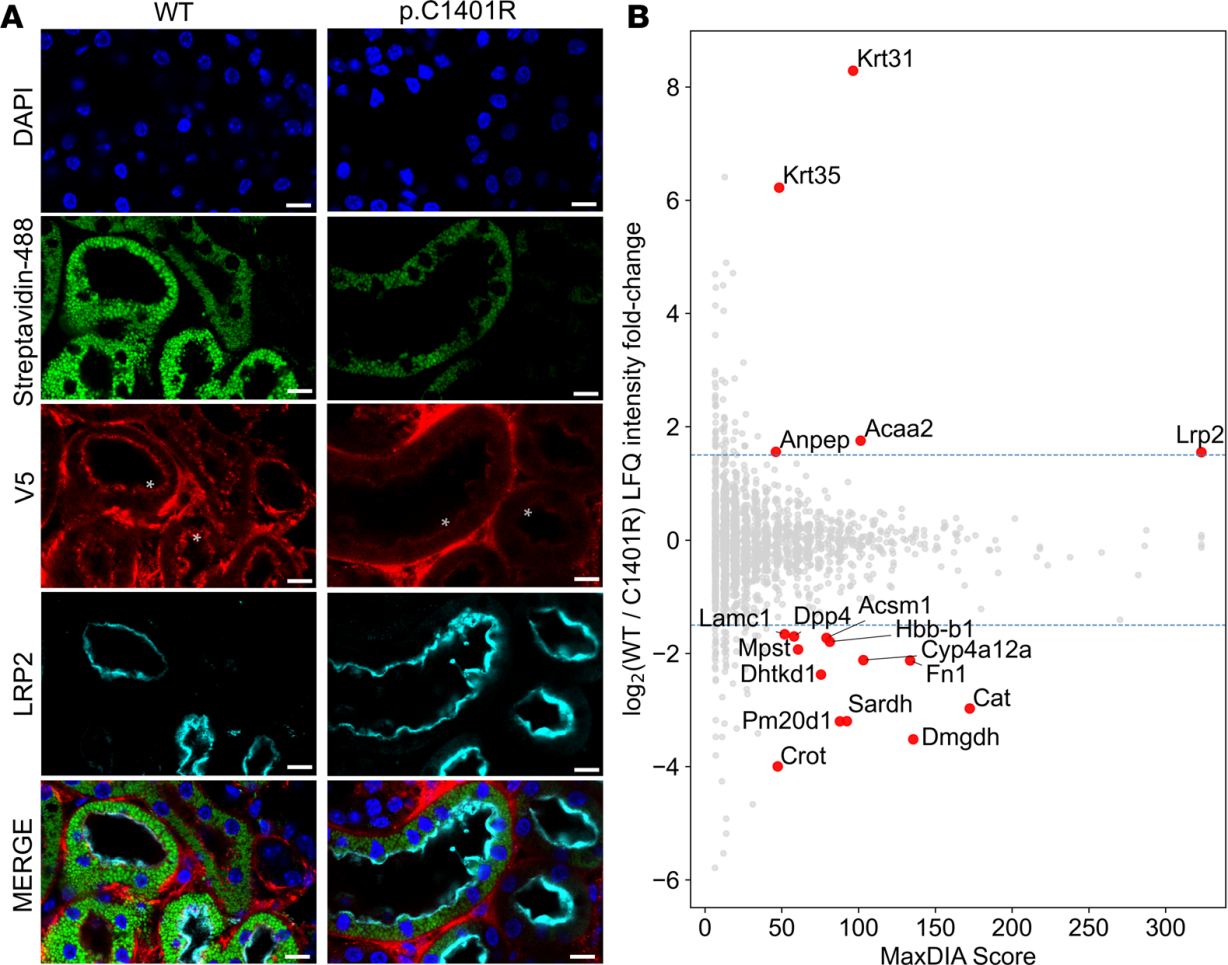
LRP2 p.C1401R and WT are differentially biotinylated by V5-APEX2-RAP. (**A**) IF of kidney sections from WT and *Lrp2* p.C1401R mice (p.C1401R) following ex vivo perfusion with the V5-tagged ascorbate peroxidase 2–receptor-associated protein (V5-APEX2-RAP) fusion construct, with labeling of biotinylated proteins with streptavidin-Alexa Fluor 488 (green), bound V5-APEX2-RAP with anti-V5 (red), and LRP2 (cyan). Gray asterisks mark selected apical membranes for comparison. Scale bars: 10 μm. Representative images from *n* = 3 mice. (**B**) Log_2_(WT/C1401R) fold change (FC) of label-free quantification (LFQ) intensities is plotted against MaxQuant data-independent acquisition analysis (MaxDIA) score for all quantified proteins. Red dots are labeled with mouse gene symbols for proteins with |log_2_FC| > 1.5 and MaxDIA score > 40. Horizontal dashed lines mark the ±1.5 log_2_FC thresholds. Key proteins are labeled, including Lrp2 showing increased abundance in WT.

IF showed clear qualitative differences between WT and *Lrp2* p.C1401R in the abundance of biotinylated apical and endocytosed proteins as indicated by streptavidin–Alexa Fluor 488 signal and anti-V5 antibody staining (Figure 5A). Differential quantitation of biotinylated proteins was accomplished with mass spectrometry and demonstrated a 2.97-fold increase in biotinylated LRP2 in WT as compared with *Lrp2* p.C1401R mice (Figure 5B). Reduced biotinylation of mutant LRP2 is consistent with its lower overall steady-state expression that was evidenced by Western blot (Figure 2, B and E) and quantitative IF (Figure 2, C and D). The presence of a signal for biotinylated LRP2 p.C1401R suggests that it retains a degree of ligand-binding affinity for RAP (Figure 5, A and B) and that the endocytic defect is likely downstream of the ligand-receptor interaction.

Despite decreased biotinylation of LRP2 p.C1401R, numerous resident proteins in the apical membrane of the mutant exhibited increased labeling by V5-APEX2-RAP, including sodium-glucose cotransporter 1 (SGLT1; log_2_ fold change [log_2_FC] –1.36) and dipeptidyl-peptidase 4 (DPP4; log_2_FC –1.70) (Table 2). It could be speculated that, in the mutant, the V5-APEX2-RAP construct remained at the apical surface longer as a result of slowed endocytosis, and thus nearby membrane proteins were labeled more extensively in the mutant compared with WT. Alternatively, the signal may represent either a true change in LRP2’s near neighbors in the mutant or possibly a change in the activity of APEX2 in the V5-APEX2-RAP fusion protein when it is bound to LRP2 p.C1401R versus WT LRP2.

### LRP2 p.C1401R perturbs endocytic flux.

**Figure 6:**
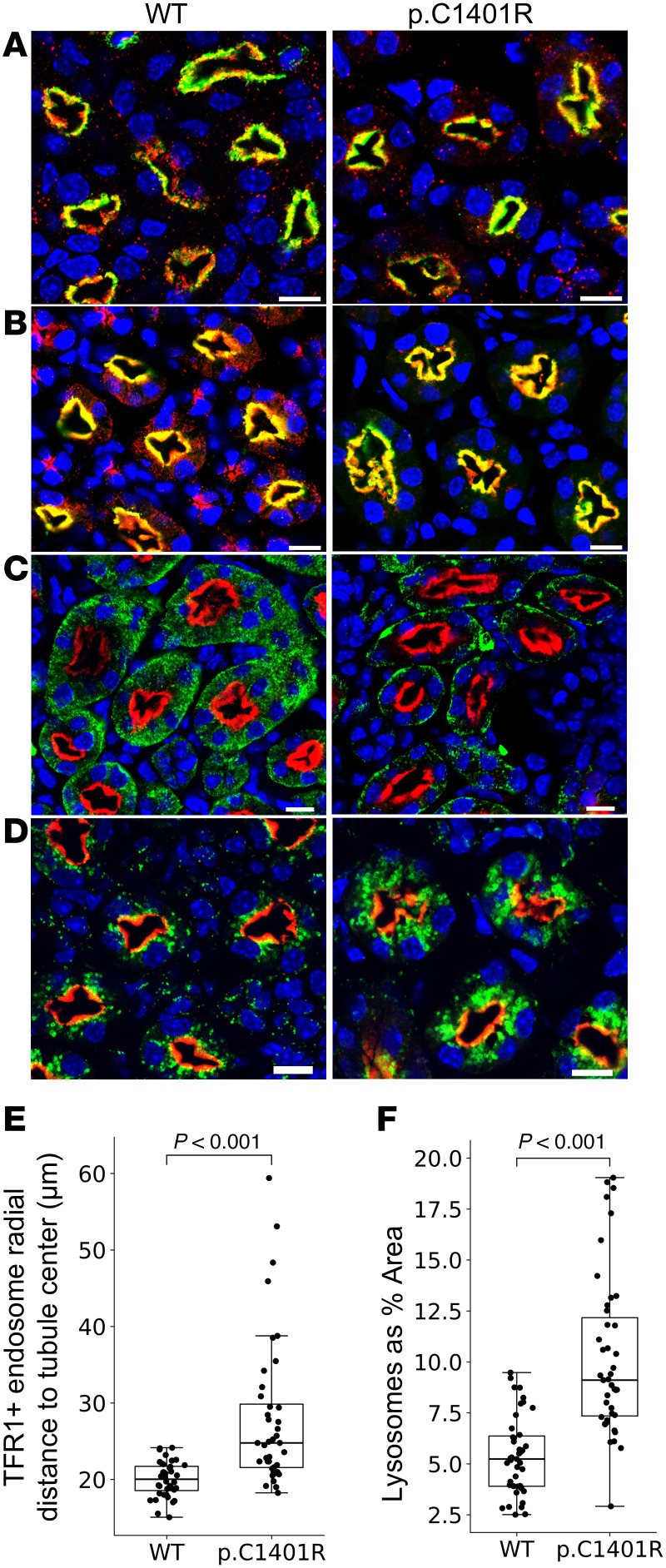
LRP2 p.C1401R perturbs endocytic flux. IF of WT and *Lrp2* p.C1401R mice (p.C1401R). (**A**) Early endosome antigen 1 (EEA1; red) relative abundance and distribution remain unchanged in *Lrp2* p.C1401R. LRP2 is in green. Scale bars: 10 μm. (**B**) RAB11 staining marking the apical recycling endosomes (AREs; red) is mislocalized in *Lrp2* p.C1401R. LRP2 is in green. Scale bars: 10 μm. (**C**) Transferrin receptor 1 (TFR1) staining marking the common recycling endosomes (CREs; green) is redistributed in *Lrp2* p.C1401R. LRP2 is in red. Scale bars: 10 μm. (**D**) Lysosomes marked by lysosomal-associated membrane protein 1 (LAMP1; green) are enlarged. LRP2 is in red. Scale bars: 10 μm. All panels are representative images from *n* = 5 mice. (**E**) Quantitation of radial distance from TFR1^+^ endosomes to the tubule center using Mann-Whitney *U* test with *N* = 40 tubule cross sections from *n* = 5 mice. (**F**) Quantitation of lysosomal area in *Lrp2* p.C1401R mice and WT mice using Mann-Whitney *U* test with *N* = 43 tubule cross sections from *n* = 5 mice. Box plots display the median (center line) and interquartile range (box boundaries), with whiskers extending to the most extreme data point within 1.5 times the interquartile range; individual data points are overlaid.

The failure of FITC-dextran uptake in the *Lrp2* p.C1401R mouse model (Figure 4C) alongside reduced apical expression of LRP2 suggests problems with PT endocytosis broader than receptor-ligand recognition. To evaluate the consequences of LRP2 p.C1401R for endocytic flux, tissue sections were immunolabeled with markers of endocytic compartments, including the early endosome with early endosome antigen 1 (EEA1), the apical recycling endosome (ARE) with RAB11, the common recycling endosome (CRE) with transferrin receptor (TFR1), and lysosomes with lysosomal-associated membrane protein 1 (LAMP1) (Figure 6, representative data from experiments with *n* = 5 mice per group) (98, 99). Probing the early endosomal compartment for EEA1 revealed an unchanged distribution (Figure 6A), but the distributions of both recycling markers RAB11 and TFR1 showed aberrant localization of these compartments, with the RAB11-labeled ARE restricted to near the apical membrane and the TFR1-labeled CRE restricted to the neighborhood of the basolateral membrane (Figure 6, B and C), reflected in a quantitative increase in the radial distance of TFR1^+^ endosomes from the tubule center in *Lrp2* p.C1401R mice (27.9 ± 9.6 μm) compared with WT (20.0 ± 2.3 μm, *P* = 7.68 × 10^–8^) (Figure 6E). Lysosomes were quantitatively enlarged in *Lrp2* p.C1401R mice (10.2% ± 5.4% of PT area) compared with WT (5.4% ± 2.0%, *P* = 4.25 × 10^–10^) (Figure 6, D and F), suggesting either a failure of membrane flux through the lysosomal compartment or increased substrate and degradation products within the lysosomes that could not be cleared.

### Structural modeling suggests that steric clashes would inhibit LRP2 p.C1401R recycling.

**Figure 7:**
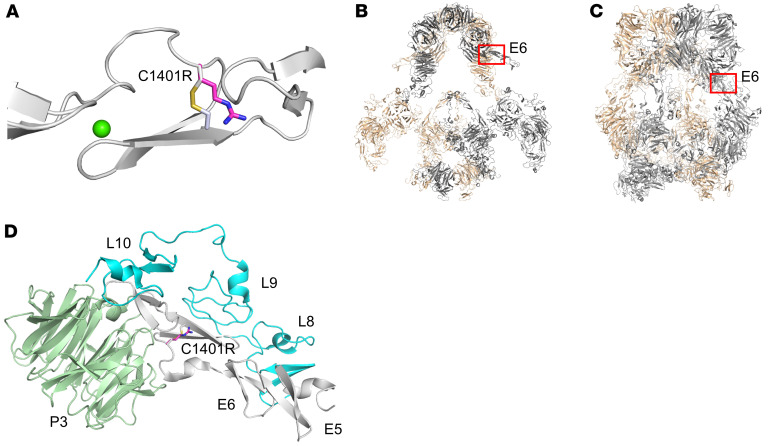
Structural modeling suggests that steric clashes would inhibit LRP2 p.C1401R recycling. (**A**) The sixth EGF-like domain (E6) is depicted in cartoon in gray, with the C1401-C1414 disulfide bridge shown, and the C1401R mutation indicated in magenta (PDB ID: 8EM4). A Ca^2+^ ion is represented as a green sphere. (**B**) The pH 7.5 LRP2 structure, with one protomer depicted in gray and the other in wheat. The position of E6 in one protomer is boxed in red. (**C**) The pH 5.2 LRP2 structure. The position of E6 in one protomer is boxed in red. (**D**) At pH 5.2 (PDB ID: 8EM7), E5 and E6 are depicted in gray, ligand-binding domains L8–L10 are in cyan, and the β-propeller P3 is in green. C1401R will likely disturb both the fold of E6 and its intramolecular interfaces.

Modeling the LRP2 p.C1401R variant in the context of the published LRP2 extracellular (pH 7.5; Protein Data Bank [PDB] ID: 8EM4) and late endosomal (pH 5.2; PDB ID: 8EM7) structures provides a framework in which to understand the finding that despite LRP2 p.C1401R’s proceeding through biosynthesis to the apical surface, endocytic flux is impaired. The cysteine-to-arginine substitution breaks a disulfide bond and introduces the large, charged side chain of arginine into the center of the fold of the sixth EGF-like repeat (E6) in LRP2. This substitution may disturb the secondary structure of an adjacent β-sheet as well as the geometry of a nearby Ca^2+^ coordination site (Figure 7A). The introduction of a free cysteine will increase the likelihood of receptor aggregation and degradation, and the disturbance within the hydrophobic core of the domain will change the overall shape of E6. The severe disturbance caused by C1401R within the E6 domain is reflected in a predicted Gibbs free energy change (ΔΔG) of 14.7 kcal/mol as calculated by Pythia (100). However, at extracellular pH, E6 is solvent-exposed and not involved in any intra- or intermolecular contacts within the LRP2 assembly (Figure 7B); thus the mutation may be tolerated during biosynthesis and trafficking to the apical surface. At the pH of the late endosome, the structure of LRP2 is more compact, and the E6 domain is buried within the assembly (Figure 7C), where it is engaged in numerous intramolecular contacts, including with ligand-binding repeats and a nearby β-propeller (Figure 7D). The disturbed fold of E6 in LRP2 p.C1401R may inhibit the formation of intramolecular interfaces between these domains and impair LRP2 from adopting its low-pH conformation required for releasing bound ligands and recycling.

## Discussion

DBS is a rare disease, yet its mechanistic consequences may offer a window into the pathophysiology of the proximal tubule in both acute and chronic kidney injury across diverse patient populations. Until recently, it has been generally regarded that LRP2 LoF leading to DBS results from failure of LRP2 expression. Endocytic defects observed in *Lrp2*-knockout mice (41), zebrafish pronephros with *lrp2* nonsense mutations (101), and CRISPR-engineered *Lrp2*-knockout opossum kidney cell lines (60) have been attributed to loss of LRP2 expression. New studies have now revealed that LRP2 harboring LoF variants can still undergo biosynthesis in model systems (55). In this study, proteomic analysis of the urine of a cohort of DBS patients led to the creation of a mouse model of the DBS variant LRP2 p.C1400R (C1401R in mouse) that has enabled the investigation of new mechanisms of PT dysfunction.

The DBS variant LRP2 p.C1401R successfully localized to the apical surface of murine PTs (Figure 2A), and a purification of the variant from mouse kidneys revealed that it can assemble into a dimer similarly to WT (Figure 3, A and B). Ex vivo perfusion of the kidneys with a V5-APEX2-RAP fusion protein to facilitate proximity biotinylation demonstrated that LRP2 p.C1401R retained a degree of ligand-binding affinity for its canonical chaperone, RAP (Figure 5, A and B). Yet despite these findings suggesting a potentially functional receptor, both receptor-mediated and fluid-phase endocytosis was significantly impaired in *Lrp2* p.C1401R mice, and IF experiments revealed strikingly restricted distributions of the markers for both common and apical recycling compartments. Remarkably, this broad range of phenotypes derived from a single amino acid substitution.

Structural modeling provides a framework for bringing these findings into congruence. LRP2 could tolerate the cysteine-to-arginine substitution at the cell surface where it resided in a solvent-exposed domain without intramolecular contacts, allowing deformation of the local domain structure without precluding the formation of LRP2’s overall dimeric assembly. However, the domain carrying C1401R becomes buried at low pH during recycling in the late endosome, and numerous intramolecular interfaces are formed. If the misshapen domain does not pack correctly, the ligand release and recycling that require LRP2’s low-pH conformation would not occur (Figure 7).

Several possibilities present themselves as to how this presumed block in receptor recycling might perturb endocytic flux globally. One hypothesis is that the misfolded receptor may sequester critical trafficking components. Analogous phenomena are well documented in neuronal systems where synaptic vesicle trafficking exhibits exquisite sensitivity to perturbations in endocytic machinery (102–105), and isolated defects can globally impair vesicle recycling as a result of the tight coupling of endocytic components (106, 107). Alternative hypotheses are suggested by the lysosomal phenotype observed in *Lrp2* p.C1401R, where the lysosomal enlargement mimics the effects of chloroquine and bafilomycin that disrupt endosomal acidification and cause trafficking failures in epithelia (108). Similar trafficking defects have been documented in renal tubular disorders such as Dent’s disease, in which mutations in *CLCN5* lead to defective endosomal acidification and endocytic dysfunction (109, 110). However, while Dent’s disease is associated with reduced apical LRP2 localization, in the *Lrp2* p.C1401R model LRP2 apical localization persists despite endocytic dysfunction.

Notably, while these findings are consistent with a recycling defect, definitive proof will require direct recycling kinetics and ligand-trapping experiments. Such experiments are necessary to prove that a recycling defect is the mechanism underlying the observed phenotype. Since these assays require surface biotinylation pulse-chase or live imaging of receptor return to the apical membrane, they will be best pursued in a knockin cellular model in future work. Nonetheless, the markedly reduced fluid-phase endocytosis in this model (Figure 4C) is most consistent with a global defect in endocytic flux and cannot be fully explained simply by reduced steady-state levels of functional protein (Figure 2). These results argue that diverse mechanisms beyond merely reduced or absent protein expression can be responsible for the phenotypes observed in patients with DBS.

The mechanistic diversity observed in DBS bears parallels to the well-characterized heterogeneity of cystic fibrosis (CF) mutations. CF mutations have been categorized into distinct classes such as channelopathies like CFTR p.G551D that impair channel gating and trafficking defects like CFTR p.F508del that prevent proper protein folding and surface localization (111, 112). These different mutations require fundamentally different therapeutic approaches (113, 114). Similarly, DBS may also be divided into functionally distinct categories. As such, this work opens new possibilities for impacting LRP2 function in the PT. Even with dimeric LRP2 expressed at the apical surface (Figure 2A and Figure 3A) that retains some degree of ligand-binding affinity (Figure 5, A and B), a general perturbation of endocytic flux occurred in the PTs of the *Lrp2* p.C1401R mouse model (Figure 6, A–F). This suggests that intervening on LRP2 to reduce its recycling may be a feasible strategy for limiting PT exposure to toxins in disease states, such as myoglobin in rhabdomyolysis.

The new reagents developed in this study have the potential for broader application. The *Lrp2* p.C1401R mouse model offers a tool with which to further dissect PT function. In a model that phenocopies the endocytic failure seen in full *Lrp2*-knockout models (2, 60), LRP2 is nonetheless expressed, enabling opportunities to examine its associations with its apical membrane partners in endocytosis, including cubilin. Additionally, the model offers an opportunity to investigate recycling machinery such as RAB11 that appears sequestered as a consequence of LRP2 dysfunction. Ex vivo proximity labeling approaches hold promise for further characterizing endocytic pathways in the kidney, especially given the specificity of a 25-minute pulse of V5-APEX2-RAP for the apical and early endosomal compartments (Table 2).

In light of this and other recent studies, it is becoming clear that DBS can result from diverse pathophysiologic pathways that converge on a similar disease phenotype. Further research on DBS-causing LRP2 missense variants will likely uncover additional structure-function relationships in LRP2 associated with LMW proteinuria. In summary, these discoveries from studies of a rare disease will expand the clinical possibilities for protecting the broader patient population from kidney injury.

## Methods

### Sex as a biological variable.

Our study examined males and females, and similar findings are reported for both sexes. Among the 9 children with DBS included in the urinary proteomics analyses, 5 were male and 4 were female, providing comparable representation for the proteinuria evaluation. A sex-stratified PCA restricted to proteins quantified across all DBS samples showed no segregation by sex along PC1 or PC2 (Figure 1D), supporting pooled analyses of male and female samples for proteinuria quantitation (Figure 1A and Supplemental Figure 2). For subsequent functional experiments in mice, female mice were used exclusively to hold sex constant across experiments.

### Human urine sample collection.

Urine samples were collected into sterile containers, and samples were centrifuged at 2,000*g* for 10 minutes at 4°C to remove cells and particulate debris. The clarified supernatants were aliquoted and stored at –80°C until proteomic analysis. Urine samples were provided by Mauro Longoni (Massachusetts General Hospital).

### Generation of Lrp2 p.C1401R knockin mice.

All animal procedures were performed in accordance with institutional guidelines and approved by the Columbia University Institutional Animal Care and Use Committee. The *Lrp2* p.C1401R knockin mouse line (homologous residue to human C1400) (Supplemental Figure 3A) was generated using CRISPR/Cas9–mediated genome editing in hybrid embryonic stem (ES) cells of the B6CBAF1 background (JAX stock 100011, The Jackson Laboratory). A C1401R/PvuI (CGATCG) mutation was introduced into exon 26 of the *Lrp2* gene by transfection of the pX459v2-*Lrp2*-80902R, which expresses the guide sgRNA-*Lrp2*-80902R and the Cas9 protein with the 100-base donor ssODN-*Lrp2*-C1401R/PvuI (ATGATACCAAGACCTGTGAAGATGTAAATGAGTGTGATATTCCAGGCTTTCGATCGCAGCACTGT-GTCAACATGAGAGGGTCCTTCCGGTGCGCTTGTGA), into hybrid B6CBA ES cells (Supplemental Figure 3B). Transfected ES cells were selected with 2 μg/mL puromycin for 24 hours and trypsinized. Five thousand trypsinized ES cells were plated out on a 15 cm feeder plate to isolate single ES colonies for screening. The genetically modified mice were generated by blastocyst injection of targeted ES cells. The PCR amplicon from targeted ES cells with the C1401R/PvuI editing can be digested by PvuI for genotyping. The WT allele yields an undigested 619 bp band, while the C1401R allele is cleaved into 336 bp and 283 bp fragments (Supplemental Figure 3C). Homozygous mice were used for all experiments except those represented in Supplemental Figure 6, in which the phenotype of heterozygous mice was also evaluated. Founder mice were maintained by sibling mating for 2 years prior to the initiation of experiments.

### Homogenization and Western blotting.

Mouse kidneys were harvested and immediately homogenized in an Eppendorf tube with Biomasher II (Kimble Chase, 749625-0030) in ice-cold homogenization buffer consisting of 150 mM NaCl (Thermo Fisher Scientific, S671), 25 mM Tris (pH 7.5) (Corning, 46-030-CM), 4% wt/vol mannitol (MilliporeSigma, M4125), 2.5% wt/vol sucrose (Thermo Fisher Scientific, S25590), 1 μg/mL aprotinin (MilliporeSigma, 10236624001), 1 μg/mL pepstatin A (MilliporeSigma, P5318), 0.5 mM PMSF (Alexis, 270-184-G005), and 5 μg/mL leupeptin (MilliporeSigma, L2884). The homogenate was spun for 10 minutes at 1,000*g* to clear cell debris, and the post-nuclear supernatant was mixed 1:1 with Laemmli sample buffer (Bio-Rad, 1610737). Protein samples were loaded alongside a prestained protein ladder (Thermo Fisher Scientific, 26619) and were separated by SDS-PAGE using a 4%–20% polyacrylamide gel (Bio-Rad, 456-1096). Proteins were then transferred onto a Nitrocellulose membrane (Bio-Rad, 162-0251) in Trans-Blot Turbo Buffer (Bio-Rad, 10026938) using the Trans-Blot Turbo Transfer System (Bio-Rad) according to the manufacturer’s instructions. After transfer, total protein loading was determined by staining with Ponceau S solution (MilliporeSigma, P-7170). The membrane was blocked with 3% bovine serum albumin (BSA; GeminiBio, 700-100P) in TBST (TBS with 0.05% Tween 20) for 30 minutes at room temperature, and then incubated overnight at 4°C with the primary antibody diluted (1:500) in 1% dry milk in TBST. Primary antibodies were LRP2/megalin (Thermo Fisher Scientific, PA5-64182; 1:500), RBP4 (Proteintech, 11774-1-AP; 1:500), LYZ (Thermo Fisher Scientific, PA5-16668; 1:500), SGLT2 (BiCell Scientific, 20802; 1:500), and CUBN (R&D Systems, AF3700; 1:500). After washing with TBST, the membrane was incubated with an appropriate HRP-conjugated secondary antibody (1:10,000 dilution) for 1 hour at room temperature. Secondary antibodies were donkey anti-rabbit IgG (H+L) (Jackson ImmunoResearch, 711-035-152; 1:10,000) and donkey anti-sheep IgG (H+L) (Jackson ImmunoResearch, 713-035-147; 1:10,000). Proteins were visualized using enhanced chemiluminescence (Kindle Bioscience, R1002) and imaged using an ImageQuant LAS4000 (GE Healthcare).

### SDS-PAGE of mouse urine samples.

Urine was collected from WT, heterozygote, and homozygote *Lrp2* p.C1401R mice (*n* = 4 per group) and spun at 10,000*g* for 15 minutes to remove cell debris, and urine loading per mouse was normalized with urine creatinine measurements (Cayman Chemical, 500701). Samples were loaded in reducing and denaturing Laemmli sample buffer and run into a 4%–20% gradient SDS-PAGE gel.

### Western blot quantitation.

Protein concentrations for gel loading were determined using the Bicinchoninic Acid (BCA) Protein Assay Kit (Thermo Fisher Scientific, 23227) according to the manufacturer’s instructions. Briefly, protein samples were mixed with BCA working reagent (reagent A:B = 50:1) and incubated at 37°C for 30 minutes, and absorbance was measured at 562 nm using a microplate reader (BioTek Synergy HTX). BSA (Thermo Fisher Scientific, 23209) was used as a standard to generate a calibration curve. Ten micrograms was loaded per lane as measured by the BCA assay.

For visual quantification of protein loading, the nitrocellulose membrane was stained following transfer with Ponceau S (MilliporeSigma, P-7170) and digitized using an Epson Perfection V300 Photo scanner. Digital images of Ponceau S–stained membranes and Western blot exposures were processed using the OpenCV computer vision library (v4) (https://opencv.org/); in a Python environment (https://www.python.org/). Lanes were detected using automatic thresholding followed by contour detection.

After lane detection, protein quantification was performed by integration of pixel intensities within each lane region. To account for membrane background and uneven staining, a local background correction method was implemented for each lane (92). Background intensity was estimated from a rectangular region immediately above each detected lane. This approach follows established practices for densitometric analysis of protein gels and blots (115).

Six biological replicates for each condition (WT or *Lrp2* p.C1401R) were analyzed on each blot, and 3 technical replicates of the blots were conducted. Data were analyzed in R in a generalized linear mixed model treating biological replicates as fixed effects, technical replicates as random effects, and Ponceau S loading controls as covariates (94).

### Immunofluorescence and immunohistochemistry.

Kidneys from WT *Lrp2* and *Lrp2* p.C1401R mice (postpartum day 6 in Figure 6C to enable staining of TFR1 [ref. 116], aged 4 months for blood urea nitrogen and creatinine measurements in Supplemental Figure 5D, otherwise aged 8 weeks for all other figures) were perfused transcardially with phosphate-buffered saline (PBS) followed by 4% paraformaldehyde (PFA) in PBS. Kidneys were then harvested, diffusion-fixed in 4% PFA for 4 hours at 4°C, cryoprotected in 30% sucrose in PBS overnight at 4°C, and embedded in OCT compound. Cryosections (5 μm) were cut and mounted. For IF staining, sections were prepared, permeabilized with 0.125% Triton X-100, blocked with 2% BSA in TBS for 1 hour at room temperature, and then incubated with primary antibodies in blocking solution overnight at 4°C. Primary antibodies were LRP2/megalin (Abcam, ab76969; 1:500), V5 (Thermo Fisher Scientific, R960-25; 1:500), EEA1 (Cell Signaling Technology, 3288; 1:100), RAB11 (Abcam, ab65200; 1:100), LAMP1 (Santa Cruz Biotechnology, sc-19992; 1:500), CUBN (R&D Systems, AF3700; 1:500), TFR1 (Bio-Rad, MCA2396; 1:100), streptavidin–Alexa Fluor 488 (Thermo Fisher Scientific, S32354; 1:500), DAPI (Thermo Fisher Scientific, 62248; 0.5 μg/mL), LTL (Thermo Fisher Scientific, L32480; 2.5 μg/mL), and SGLT2 (Abcam, ab306558; 1:100). Sections were washed in PBS 3 times for 5 minutes, and then incubated with secondary antibodies for 1 hour at room temperature. Secondary antibodies were donkey anti-rabbit IgG antibody Alexa Fluor 647 (Jackson ImmunoResearch, 711-606-152; 1:500), donkey anti-mouse IgG antibody Alexa Fluor 594 (Jackson ImmunoResearch, 715-586-151; 1:500), and donkey anti-sheep IgG antibody Alexa Fluor 594 (Jackson ImmunoResearch, 713-585-147; 1:500). Sections were then washed 6 times for 5 minutes each and then mounted with VECTASHIELD PLUS Antifade Mounting Medium (Vector Laboratories, H-1900-10). Tissue was imaged using a Leica SP8-DLS confocal microscope, and images were processed in ImageJ (NIH).

### Quantification of immunofluorescence.

The circumference of LRP2^+^ tubules was traced in ImageJ. The outside of the region of interest (ROI) was cleared, and the inside of the ROI was thresholded using the Bernsen method to identify LAMP1^+^ lysosomes or myoglobin^+^ endosomes. The total area of identified structures was then quantified using the Analyze Particles tool with minimum size = 0.3 and circularity = 0.0–1.00. The percentage lysosomal or myoglobin^+^ endosomal area was then calculated by dividing the total lysosomal or endosomal area by the total ROI.

For TFR1^+^ endosomal distribution, the centroids of TFR1^+^ endosomes were identified by thresholding using the Bernsen method with radius = 50 followed by the Analyze Particles function with objects less than 0.05 μm**^2^** in area filtered out. The center of the tubule was derived by the same method using the centroid of LRP2 staining (Bernsen, radius = 50). The radial distance was then calculated between each endosome and the center of the tubule and averaged to yield the average radial distance of TFR1^+^ endosomes to the tubule center in micrometers.

For LRP2 IF quantitation, cryostat sections of *Lrp2* WT, *Lrp2* p.C1401R, *Lrp2*^5′CreERT2/+^, and *Lrp2*^5′CreERT2/fl^ mice were air-dried for 2 hours, rehydrated in PBS, and blocked with 2% BSA/0.125% Triton X-100 in PBS for 1 hour at room temperature. Sections were incubated for 2 hours at room temperature with directly conjugated anti-LRP2–Alexa Fluor 594 (Novus Biologicals, NB110-96417AF594; 1:500) and anti-ezrin (Thermo Fisher Scientific, MA5-13862; 1:200). After 3 washes in blocking buffer, Alexa Fluor 488–conjugated goat anti-mouse secondary antibody (Jackson ImmunoResearch, 115-545-205; 1:500) was applied to detect ezrin. Slides were mounted and imaged by confocal microscopy.

LRP2^+^ membrane area was quantified in ImageJ. Proximal tubule cross sections were delineated using the ezrin costain by tracing of the tubule circumference with the freehand ROI tool. Within each ROI, LRP2 signal was segmented by auto-thresholding using the Bernsen method (radius = 15). LRP2^+^ structures were identified using the Analyze Particles function with a minimum size filter of 0.1 μm^2^. The summed area of LRP2^+^ particles was divided by the total tubule cross-sectional area to yield the normalized LRP2^+^ fractional area.

For all quantitations, 9 fields of view were evaluated from 3 separate mice, and data from the 40–50 tubules in these fields of view were aggregated. Data were collected at ×60 original magnification with 2,048 × 2,048 pixel resolution.

### Endocytosis assays.

Mice were intraperitoneally injected with FITC-dextran (Invitrogen, D1820; 0.25 mg in 100 μL PBS per mouse), Alexa Fluor 594–labeled BSA (Thermo Fisher Scientific, A13101), or Alexa Fluor 568–labeled myoglobin, prepared by labeling of equine skeletal muscle myoglobin (MilliporeSigma, M0630) with Alexa Fluor 568 NHS Ester (Invitrogen, A20003) according to the manufacturer’s instructions. After 30 minutes, mice were anesthetized, and kidneys were perfused with PBS followed by 4% PFA, harvested, and processed for cryosectioning as described above.

### Serum blood urea nitrogen and creatinine measurements.

Whole blood was collected from the ophthalmic vein from *n* = 4 mice per genotype at 4 months of age and analyzed immediately using i-STAT CHEM8+ cartridges (Abbott, 09P31-26) to determine blood urea nitrogen and creatinine.

See Supplemental Methods for details regarding urinary biomarker measurements; RNAscope; size exclusion chromatography analysis; OptiPrep density gradient equilibrium ultracentrifugation; cloning and production of V5-APEX2-RAP; perfusion of recombinant V5-APEX2-RAP in WT and *Lrp2* p.C1401R mouse kidneys; isolation of biotinylated proteins; MS analysis of the urinary proteome; MS analysis of V5-APEX2-RAP–labeled mouse kidneys; MS analysis of tryptic peptides from LRP2 p.C1401R; and structural modeling.

### Statistics.

Proteomics intensity data were processed using Python 3 with the following libraries for data management and statistical analysis: pandas (v1.5.3), NumPy (v1.24.3), SciPy (v1.10.1), and statsmodels (v0.13.5). Plotting was conducted with Matplotlib (v3.7.1). Raw intensity values from MS experiments were imported from Excel-format files, and log_2_-transformed differential protein expression data between control and experimental groups were assessed using Welch’s 2-tailed *t* test, which does not assume equal variances between groups. Only proteins with at least 2 valid intensity measurements in each group were included in the statistical analysis. *P* values were adjusted for multiple testing using the Benjamini-Hochberg false discovery rate (FDR) method with an α threshold of 0.01 (117). A *q* value less than 0.01 was considered significant.

Proteins with missingness that precluded Welch’s *t* test were evaluated by Fisher’s exact test for categorical differential presence of quantifiable proteins between DBS patients and controls. For each protein, a 2 × 2 contingency table was constructed comparing the frequency of detection (non-missing intensity values) between control and experimental groups. The resulting *P* values were again corrected for multiple testing using the Benjamini-Hochberg procedure at FDR < 0.01. Proteins were classified as enriched in either the control or experimental group based on their relative detection frequencies.

Dimensionality reduction was performed using principal component analysis (PCA) as implemented in scikit-learn (v1.2.2). A complete-case analysis approach was used, including only proteins with intensity measurements across all samples to avoid imputation bias. The intensity matrix was mean-centered by protein before decomposition.

For statistical comparisons of IF quantifications, either Welch’s 2-sample *t* test (2-tailed) was used when both groups were approximately normally distributed by the Shapiro-Wilk test (myoglobin data in Figure 4B), or the Mann-Whitney *U* test (2-sided) was used for non-parametric data that failed the Shapiro-Wilk test (TFR1^+^ endosome data in Figure 6E and lysosome data in Figure 6F). A *P* value less than 0.05 was considered significant. Box plots display the median (center line) and interquartile range (box boundaries), with whiskers extending to the most extreme data point within 1.5 times the interquartile range; individual data points are overlaid.

For proximity biotinylation data, label-free quantification (LFQ) intensities were extracted for both LRP2 p.C1401R and WT LRP2 samples. Only proteins with valid LFQ intensity measurements in both WT LRP2 and LRP2 p.C1401R conditions and a valid MaxQuant data-independent acquisition analysis (MaxDIA) score were retained for downstream analysis. In addition to application of a global FDR filter of 1%, a MaxDIA score threshold of 40 was used to stratify the results for the proteins with multiple high-quality peptide identifications (118).

### Study approval.

This study involved 9 children diagnosed with clinical DBS based on characteristic clinical features and confirmed pathogenic variants in LRP2 (Table 1; Supplemental Figure 1; and Frances A. High and Patricia K. Donahoe, unpublished data) alongside 8 healthy parental controls who were unsequenced but are presumed carriers. The research protocol was conducted in accordance with IRB review (IRB no. 2009P001589, Partners HealthCare System, Boston, Massachusetts). Written informed consent was obtained from the parents or legal guardians of all participating children and from all adult controls prior to their inclusion in the study.

Mice were maintained in the Columbia University Irving Medical Center Barrier Facility under specific pathogen–free conditions. The facility is accredited by the Association for Assessment and Accreditation of Laboratory Animal Care International and operates in compliance with the *Guide for the Care and Use of Laboratory Animals* (National Research Council; National Academies Press, 2011) and applicable federal regulations (USDA and NIH). All experimental procedures were approved by the Columbia University Institutional Animal Care and Use Committee.

### Data availability.

Data values for figures are provided in the Supporting Data Values file. The raw mass spectrometry data can be retrieved at the Mass Spectrometry Interactive Virtual Environment (MassIVE) with accession codes MSV000098796 and MSV000098798. The *Lrp2* p.C1401R mouse has been deposited at The Jackson Laboratory as JAX strain ID 419745. The V5-APEX2-RAP bacterial expression construct has been deposited at Addgene as plasmid 241358. Sequencing data for the DBS patients characterized in the study are available at the NCBI’s Database of Genotypes and Phenotypes (dbGaP) under accession code phs000783.v2.p1.

## Author contributions

AB and JB conceived the study. AB, THS, AG, HEB, FAH, JL, JSK, RES, JRA, LDS, GHR, and LK conducted experiments. AB, TS, AG, HEB, JSK, PKD, TAN, FAH, OAW, and JB contributed to experimental design. AB, AG, HEB, AK, and TAN performed data analysis and visualization. AB, JB, TAN, JSK, AK, PKD, FAH, and OAW provided funding. AB and JB wrote the manuscript. All authors reviewed and edited the manuscript.

## Conflict of interest

The authors have declared that no conflict of interest exists.

## Funding support

This work is the result of NIH funding, in whole or in part, and is subject to the NIH Public Access Policy. Through acceptance of this federal funding, the NIH has been given a right to make the work publicly available in PubMed Central.

- NIH R01 DK124667 (to JB).
- NIH U54 DK104309 (to JB).
- NIH R01 DK125049 (to OAW).
- NIH R01 HD115718 (to FAH).
- NIH P01 HD068250 (to PKD).
- NIH S10 RR027990 (to TAN).
- NIH K08 DK132511 (to AB).
- NIH K25 DK128563 (to AK).
- NIH K08 HL151969 (to JSK).
- American Heart Association Career Development Award 35320208 (to JSK).
- Gerstner Family Foundation (to AB).
- American Society of Nephrology KidneyCure Carl W. Gottschalk Scholar Award (to AB).
- Massachusetts General Hospital Executive Committee on Research (to FAH).
- Roy and Diana Vagelos Precision Medicine Award (to AB and JB).

## Supplementary Material

Supplemental data

Unedited blot and gel images

Supporting data values

## Figures and Tables

**Table 1:**
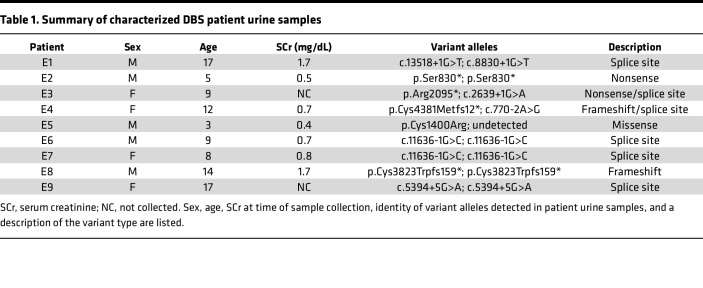
Summary of characterized DBS patient urine samples

**Table 2:**
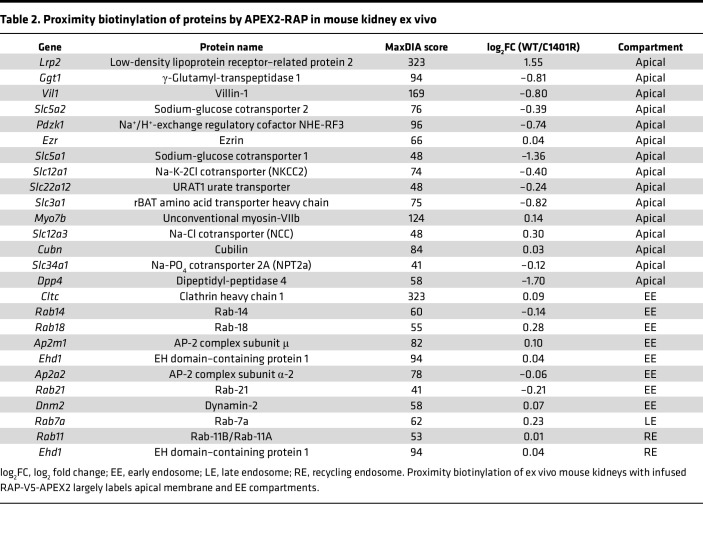
Proximity biotinylation of proteins by APEX2-RAP in mouse kidney ex vivo

## Version 1 04/23/2026

In-Press Preview

## Version 2 06/22/2026

Electronic publication
