# Supplementary material for: Failure of endocytic flux in Donnai-Barrow syndrome caused by LRP2 p.C1400R
Source: JCI Insight. 2026 Apr 23;11(12):e199341. doi: 10.1172/jci.insight.199341 (PMC13313491; doi:10.1172/jci.insight.199341)
Supplement: Supplemental data [file jciinsight-11-199341-s096.pdf]

**Supplemental Material**

**Supplemental Methods**

**Urinary biomarker measurements**

Urine was collected from mice by manual bladder expression and stored at −80°C until analysis. Urinary creatinine was measured using the Creatinine Colorimetric Assay Kit (Cayman Chemical, 500701). Urinary albumin was quantified using the Mouse Albumin ELISA Kit (Bethyl Laboratories, E99-134). Urinary KIM-1 (*Havcr1*) was measured using the Mouse KIM-1 ELISA Kit (Abcam, ab213477). All assays were performed according to the manufacturers' instructions. Albumin and KIM-1 concentrations were normalized to urinary creatinine concentration and expressed as urinary albumin-to-creatinine ratio (UACR, mg/g) and KIM-1-to-creatinine ratio (ng/mg).

**RNAscope**

In situ hybridization on formalin-fixed paraffin-embedded (FFPE) mouse kidney tissue was performed using the chromogenic RNAscope 2.5 HD duplex reagent kit (ACDbio, 322430) according to the manufacturer’s protocols. Briefly, tissue sections were deparaffinized and rehydrated with xylene and ethanol washes, blocked with hydrogen peroxide, heated in target retrieval buffer, and digested by protease. The tissue sections were then incubated with the Mm-Havcr1 RNAscope target probe (Biotechne, 472551) undiluted at 40°C in a hybridization oven for 2 hours. RNA signal was amplified using the kit’s amplification system conjugated to horseradish peroxidase (HRP), and signal was detected using a chromogenic substrate solution. Tissue sections were then counterstained with hematoxylin, dried in a 60°C oven, and mounted for image analysis. Bright-field images were captured using the Olympus IX73 inverted microscope 20x objective.

**Size exclusion chromatography analysis**

LRP2 WT and LRP2 p.C1401R were purified as previously described (61), and post-anion exchange LRP2-containing fractions were pooled, brought to 0.5mL using Amicon concentrators

(Millipore #UFC210024), and then injected over a Superose 6 10/300 (Cytiva, #17517201) size-exclusion column equilibrated in 150 mM NaCl, 0.8% OG, 25 mM Tris pH 7.5.

**Optiprep Density Gradient Equilibrium Ultracentrifugation**

0.5mL of PNS prepared from mouse kidney homogenate was loaded on top of a step gradient consisting 2mL each of 5, 10, 15, 20, 25, and 30% Optiprep medium (Sigma, #D1556) in 0.25M sucrose, 10mM Hepes pH 7.5 with a 1mL 60% cushion at the base in Beckman Open-Top Thinwall Ultra-Clear 14 mL ultracentrifuge tubes (Beckman, #344060). The sample was spun in a SW 40 Ti rotor at 22,000 rpm (85,852g) for 16hrs on a L7 Ultracentrifuge (Beckman), during which time the step gradient linearizes. 1mL fractions were unloaded from the gradient with a Gradient Station (BioComp). Samples were loaded by equal volume to enable direct comparison of the distribution of cell compartment markers.

**Cloning and production of V5-APEX2-RAP**

The BamHI site was used to insert V5-APEX2 with a linker into the human RAP construct in pGEX-2T(Supplemental Figure 8A)(Dudley Strickland, University of Maryland, Baltimore, MD) (1). The insertion orientation and sequence of the V5-APEX2-RAP fusion construct were verified by Sanger sequencing with primers, 5GEX GGGCTGGCAAGCCACGTTTGGTG and 3GEX CCGGGAGCTGCATGTGTCAGAGG. Recombinant protein was induced by 1mM IPTG in BL21 E. coli, purified by GST affinity and eluted with PBS following on-column thrombin digestion (Supplemental Figure 8B). After LPS extraction with Triton X-114, V5-APEX2-RAP was sized on a Sephadex 75 sizing column prior to use in mice.

**Perfusion of Recombinant V5-APEX2-RAP in WT and *Lrp2* p.C1401R Mouse Kidneys.**

Kidneys were harvested from a wild type control mouse and *Lrp2* p.C1401R mouse according to approved Columbia IACUC protocols. All mice were injected with 5000 U/kg of Heparin Sodium (25021-400-30, Sagent Pharmaceuticals) i.p. five minutes before euthanasia to improve renal perfusion post-mortem. After removing fat and exposing the renal artery, the kidney was immersed into perfusion solution (NaCl 141.8 mmol/L, KCl 4.7 mmol/L, MgSO4 1.7 mmol/L,

EDTA 0.5 mmol/L, CaCl2 2 mmol/L, HEPES 10 mmol/L, KH2PO4 1.2 mmol/L, glucose 5 μmol/L). All perfusion solution chemicals were obtained from Sigma Aldrich. Perfusion was carried out at constant pressure of 60mmHg generated with a peristaltic pump (PS200, Living Systems Instrumentation) in a blood vessel perfusion chamber (CH1, Living Systems Instrumentation) following cannulation of the renal artery. Kidney was first perfused with 25 nM V5-APEX2-RAP, 0.5 mM biotin-phenol in the above perfusion solution, with at least 5 ml of 0.5 mM biotin-phenol passing through the kidney. Next, the buffer was switched to 0.3% H2O2 freshly prepared in PBS for 5 min, followed by 25 min perfusion with ice cold quenching buffer (10 mM sodium ascorbate, 10 mM sodium azide, in PBS). The kidney was bisected, with one half frozen at -80 °C for subsequent proteomics and the other fixed in 4% PFA for immunostaining.

**Isolation of Biotinylated Proteins**

For proteomics, the half kidney was homogenized in 900 ul of TEAB buffer without SDS (125 mM TEAB, 75 mM NaCl with both phosphatase and protease inhibitor tablets) using a sterile, disposable pestle. Subsequent to homogenization, 100 ul 10% SDS was added (final 1% SDS), and the homogenate was mixed and incubated on ice for a further 15 min. The lysate was sonicated with 10 pulses of 10 sec each on ice with 10 sec pause between each pulse with a Branson Sonifier 450 at output level 3, and then cleared by centrifugation at 10,000g at 4°C for 15 min. 300 μg of protein was used, volume was adjusted to 200 ul with TEAB buffer without SDS, 20 ul of 0.2 M DTT was added, and the protein mixture was incubated at RT with rotation for 30 min. Then, 60 ul of freshly prepared 0.2 M iodoacetamide was added and incubated at RT for a further 30min, protected from light. The treated protein mixture was incubated with 150 ul streptavidin magnetic beads at RT with rotation for 90 min. The beads were sequentially washed with 0.5 ml TEAB buffer without SDS, 0.5 ml 1 M KCl, and then 5 times with 0.5 ml of

0.1 M TEAB. Tubes were changed after each wash, and the beads were frozen with 30 ul 0.1 M TEAB buffer at -80 °C before mass spectrometry analysis.

**Mass spectrometry analysis of the urinary proteome**

Gel bound samples for patients and controls were processed for in-gel digestion using trypsin (trypsin gold, mass spectrometry grade, Promega) protease. The peptides generated were cleaned up with in-house optimized Empora C18 High Performance Extraction Disks (3M, MN). Peptide pools were subjected to nano liquid chromatograpy coupled to tandem mass spectrometry (nano LC-MS/MS) using a Thermo Fisher Q-Exactive HF instrument operated in data-dependent-analysis (DDA) mode as described (2). All mass spectra were first converted to mgf peak list format using Proteome Discoverer 1.4 and the resulting mgf files searched against a human Uniprot protein database (*Homo sapiens* TaxId = 9606, UniProtKB release 2019_11, 20605 proteins) using Mascot (Matrix Science, London,UK; version 2.5.0). Decoy proteins were added to the search to allow for the calculation of false discovery rates (FDR). The search parameters were as follows: (i) up to two missed tryptic cleavage sites were allowed; (ii) precursor ion mass tolerance = 10 ppm; (iii) fragment ion mass tolerance = 0.8Da; and (iv) variable protein modifications were allowed for methionine oxidation, deamidation of asparagine and glutamines, and protein N-terminal acetylation. MudPit scoring was applied using significance threshold score $p<0.01$. Decoy database search was always activated and, in general, for merged LS-MS/MS analysis of a gel lane with $p<0.01$, false discovery rate averaged around 1%. The Mascot search result was finally imported into Scaffold (Proteome Software, Inc., Portland, OR; version 4.7.3) to further analyze tandem mass spectrometry (MS/MS) based protein and peptide identifications. The search engine results were combined and displayed at 1% FDR. Protein and peptide probability was set at 95% with a minimum peptide requirement of 2. Protein identifications were expressed as Exclusive Spectrum Counts that identified each protein listed.

**Mass spectrometry analysis of V5-APEX2-RAP labeled mouse kidneys**

Protein relative abundances were determined by label-free relative quantitative proteomics (MaxLFQ) using data-independent acquisition (MaxDIA) (3). Magnetic bead bound Wild Type LRP2 (WT) and C1401toR LRP2 (CR) -APEX samples were subjected to on-bead reduction and alkylation of cysteine residues, followed by rinses with 50mM NH4HC$O_3$. On-bead trypsin (mass spectrometry grade Trypsin Gold, Promega, Madison, WI, USA) at 5ng/μl in 50 mM $NH_4HCO_3$ was used for overnight digestion. After acidification with 10% formic acid, peptides were extracted with 5% formic acid/50% Acetonitrile (v/v) and concentrated to a small droplet using vacuum centrifugation. Desalting of peptides was done using hand packed SPE Empore C18 Extraction Disks (3M St.Paul, MN, USA) as described (4). Desalted peptides were again concentrated and reconstituted in 10 μL 0.1% formic acid in water. Aliquots (5uL) of the peptides were analyzed by nanoflow liquid chromatography followed by tandem mass spectrometry (nano-LC-MS/MS) using an Easy nLC 1000 equipped with a self-packed New Objective 75 μm x 20 cm reverse phase column (packed with ReproSil-Pur C18, 3 μm beads, Dr. Maisch GmbH, Germany) coupled online to a QExactive HF Orbitrap mass spectrometer via a Nanospray Flex source (all instruments from Thermo Fisher Scientific, Waltham, MA, USA). Analytical column temperature was maintained at 50ºC by a column oven (Sonation GmBH, Germany). Peptides were eluted with a 3-40% acetonitrile gradient over 110 min at a flow rate of 250 nL/min. The mass spectrometer was operated in data-independent acquisition mode; MS survey scans were acquired in profile mode, at a resolution of 120,000 (at *m/z* 200) over a scan range of 300-1650 *m/z*. Following the survey scans, 30 groups of precursors were selected for fragmentation with sliding isolation windows to include peptide *m/z* values ranging from 364 to 1370 Th. The default maximum charge state was set to 4 and resolution was set to 30,000. In MS/MS, the fixed first mass was set to 200 Da. The normalized collision energy (NCE) /stepped NCE was 25.5,27,30. The maximum injection times for the survey scan was 60 ms and for MS/MS, it was set to auto. The ion target value for both scan modes was set to 3e6. Data were analyzed by the software MaxQuant (version 2.5.1.0), referred to as MaxDIA analysis

type. To identify peptides, we used mouse in silico-generated spectral libraries that were created from the Uniprot mouse protein sequence database (downloaded on 06/18/2020; 21,989 entries). The library was obtained from the Max Plank Institute of Biochemistry Data share drive (https://datashare.biochem.mpg.de). After MaxDIA analysis, the proteinGroups matrix was imported into Perseus (version 1.5.6.0, (5)). We carried out imputation of missing values by random numbers that are drawn from the normal distribution of the overall data (width=0.3; downshift=1.8). To determine the relative abundance of a protein across a set of samples, we used its label free quantitation (MaxLFQ) intensity calculated by MaxDIA, which normalizes the protein intensity by normalizing peptide intensities across samples.

**Mass spectrometry analysis of tryptic peptides from LRP2 p.C1401R**

ThermoFisher Scientific XCalibur (version 4.1.31.9) software was used to create layouts containing predicted experimental masses with a mass accuracy set to 5ppm, and total ion current of the MS1 spectra was manually extracted at the predicted, doubly charged monoisotopic masses in the wild type and mutant spectra. A proteomics tool, MS-Tag, developed in the UCSF Mass Spectrometry Facility (prospector.ucsf.edu/prospector/mshome.html) was used to predict y ion series (C-terminal directed) and b ion series (N-terminal directed) of the fragmented peptide (MS/MS spectrum).

**Structural Modeling**

LRP2 at pH 7.5 (PDB ID: 8EM4) and pH 5.2 (8EM7) were visualized with PyMOL 2.3.4 (Schrödinger, LLC). ΔΔG was estimated using Pythia (6) with the sequence for E6 from mouse LRP2 (Uniprot ID: A2ARV4), residues 1391-1430.

1. Williams SE, et al. A novel mechanism for controlling the activity of alpha 2-macroglobulin receptor/low density lipoprotein receptor-related protein. Multiple regulatory sites for 39-kDa receptor-associated protein. J Biol Chem. 1992;267(13):9035–40.
2. Xu Y, et al. Cardiolipin remodeling enables protein crowding in the inner mitochondrial membrane. EMBO J. 2021;40(23):e108428.
3. Sinitcyn P, et al. MaxDIA enables library-based and library-free data-independent acquisition proteomics. Nat Biotechnol. 2021;39(12):1563–73.
4. Rappsilber J, et al. Protocol for micro-purification, enrichment, pre-fractionation and storage of peptides for proteomics using StageTips. Nat Protoc. 2007;2(8):1896–906.
5. Tyanova S, et al. The MaxQuant computational platform for mass spectrometry-based shotgun proteomics. Nat Protoc. 2016;11(12):2301–19.
6. Sun J, et al. Structure-based self-supervised learning enables ultrafast protein stability prediction upon mutation. Innovation (Camb). 2025;6(1):100750.

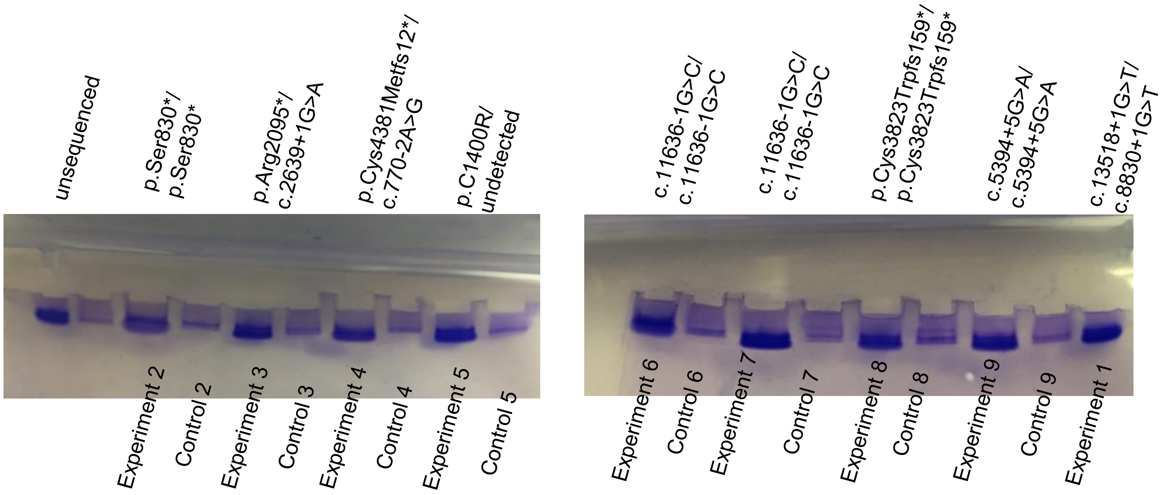


**Supplemental Figure 1: Experimental and Control Samples for Proteomic Analysis.** SDS-PAGE reducing gels of experimental samples from children with DBS and control samples from their mothers. Protein samples were briefly run into the gel for visualization. Above the gel, genotypes of the patient samples are indicated. Mothers were not genotyped and are presumed heterozygous carriers. Experiments 6 and 7 share the same genotype and are siblings. Two aberrant LRP2 alleles were detected for each patient except for the patient associated with Experiment 5, who did not have a second variant allele detected. Below the gel, each sample's corresponding experiment or control is listed. A patient with clinical DBS but who was lost to follow-up and not sequenced was included on the gel but not included in the proteomic analysis.

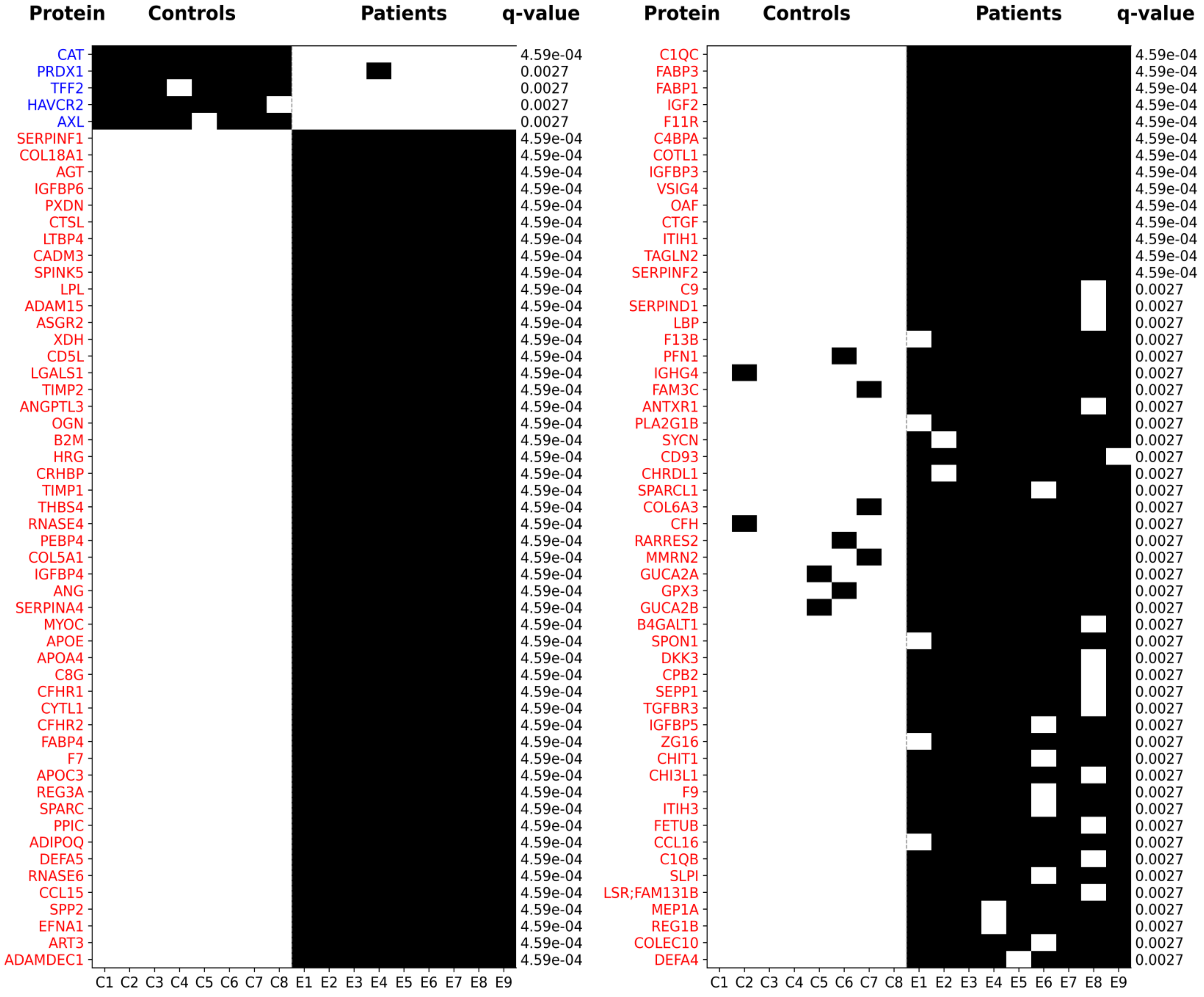


**Supplemental Figure 2: Proteomics of Donnai-Barrow Syndrome**

For proteins not detected in enough samples across both groups to conduct Welch's t-test, a Fisher's Exact Test was conducted with BH-FDR correction q<0.01, identifying five significant proteins in controls (blue), and 105 significant proteins in patients (red). A black box identifies a patient or control with quantifiable intensity for the listed protein; white boxes have no quantifiable intensity for the listed protein. Proteins are listed along the y-axis and patients (E1-9) and controls (C1-8) on the x-axis.

A

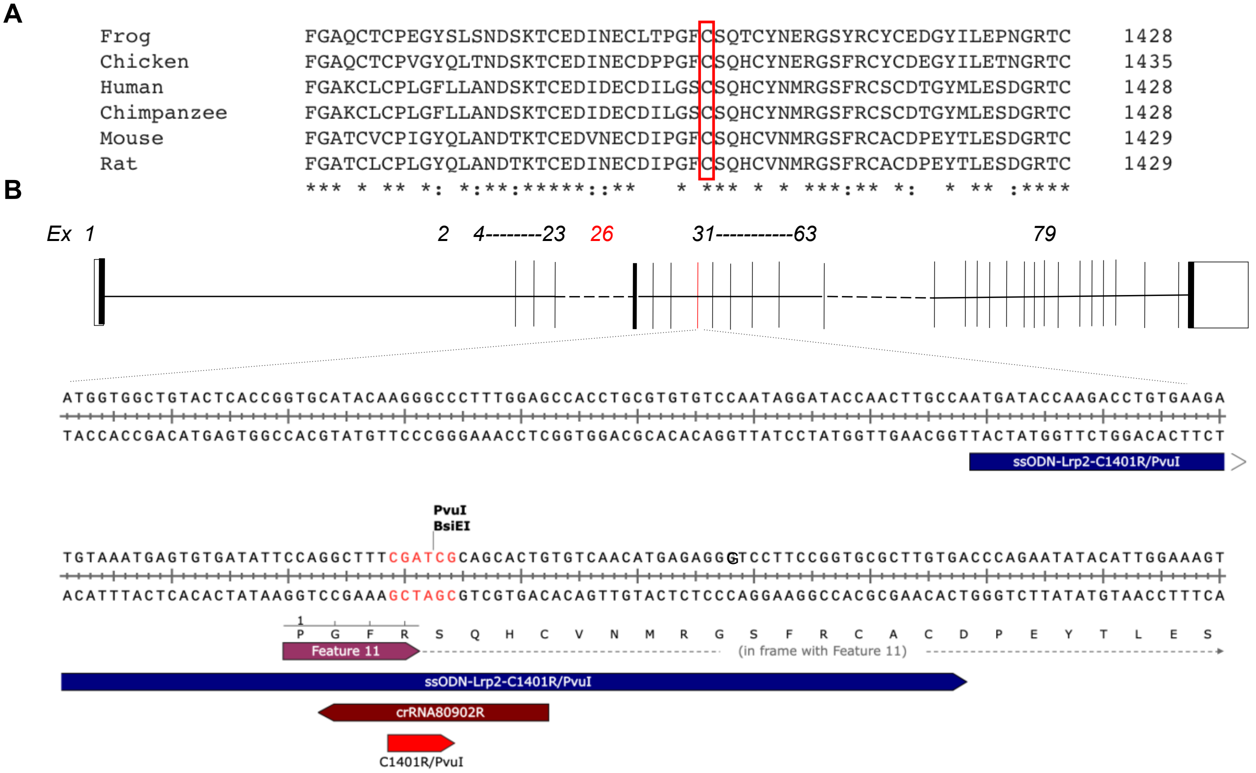


C

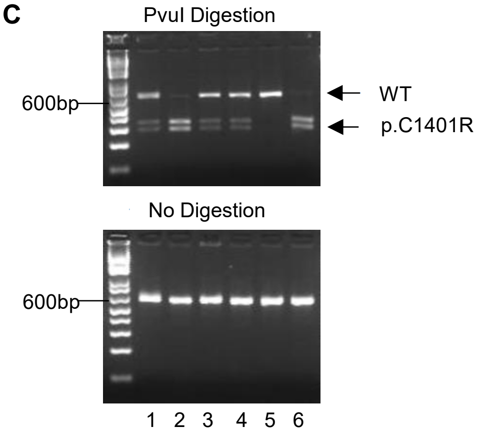


**Supplemental Figure 3: CRISPR mouse model *Lrp2* p.C1401R to model the DBS variant LRP2 p.C1400R. (A)** Clustal sequence alignment of Lrp2 protein sequences from amphibians, avians, and mammals. The conserved cysteine at position 1400 in human is boxed in red along with the homologous residues in other species. "*" indicates an identical amino acid; ":" indicates a similar amino acid. **(B)** Generation of *Lrp2 p.C1401R* allele*:* A C1401R mutation incorporating a silent PvuI site (CGATCG) was introduced into exon 26 of *Lrp2* by transfecting pX459v2-*Lrp2*-80902R which expresses the guide sgRNA-*Lrp2*-80902R and the Cas9 protein with ssODN-*Lrp2*-C1401R/PvuI. **(C)** Genotyping of *Lrp2*$^{C1401R/C1401R}$: After PvuI digestion of a 619bp PCR product, the C1401R allele yields a 336 bp and 283 bp double band, while the WT allele remains undigested. Thus, lanes 2 and 6 are *Lrp2*$^{C1401R/C1401R}$, lanes 1, 3, 4 are *Lrp2*$^{C1401R/+}$, lane 5 is *Lrp2*$^{+/+}$.

A

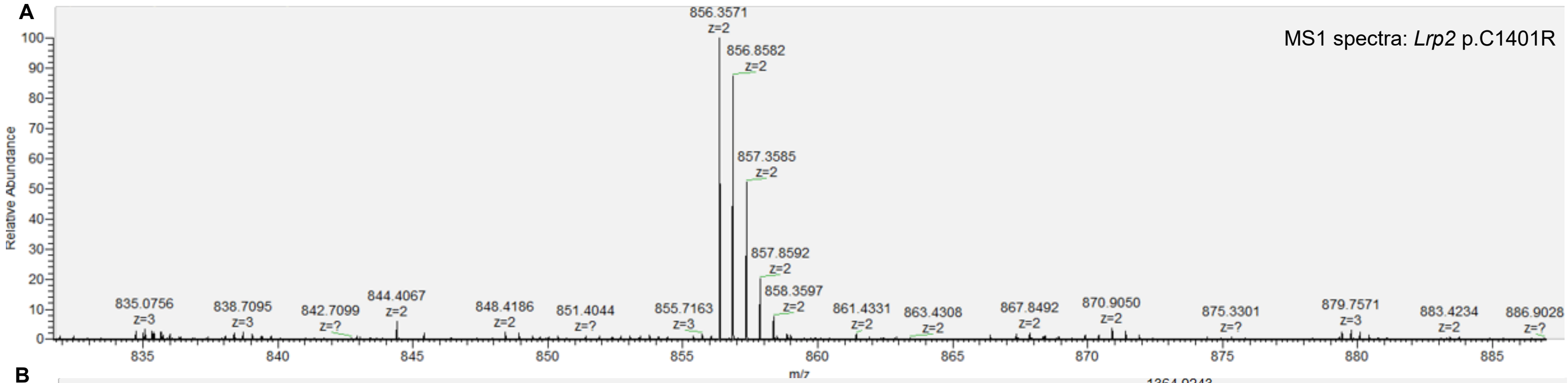


B

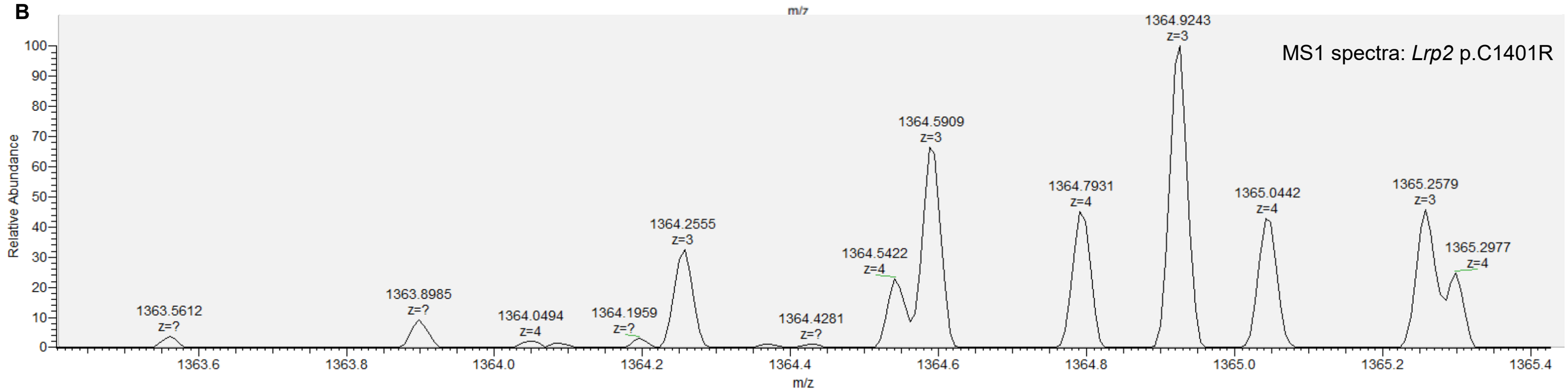


C

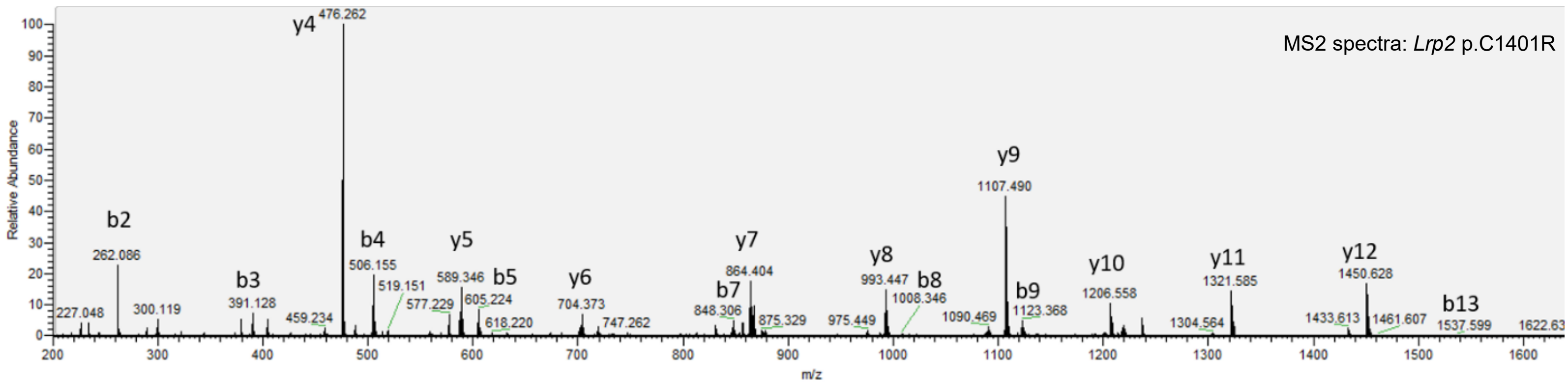


D

| b | | | | y |
|---|---|---|---|---|
| --- | 1 | T | 14 | --- |
| 262.0856 | 2 | C(Carbamidomethyl) | 13 | 1610.6574 |
| 391.1282 | 3 | E | 12 | 1450.6267 |
| 506.1551 | 4 | D | 11 | 1321.5841 |
| 605.2236 | 5 | V | 10 | 1206.5572 |
| 719.2665 | 6 | N | 9 | 1107.4888 |
| 848.3091 | 7 | E | 8 | 993.4458 |
| 1008.3397 | 8 | C(Carbamidomethyl) | 7 | 864.4032 |
| 1123.3667 | 9 | D | 6 | 704.3726 |
| 1236.4507 | 10 | I | 5 | 589.3457 |
| 1333.5035 | 11 | P | 4 | 476.2616 |
| 1390.5250 | 12 | G | 3 | 379.2088 |
| 1537.5934 | 13 | F | 2 | 322.1874 |
| --- | 14 | R | 1 | 175.1190 |

**Supplemental Figure 4: Mass spectrometry detection of mutant but not WT peptide in purified *Lrp2* p.C1401R. (A)** The tryptic peptide for *Lrp2* p.C1401R, TCEDVNECDIPGF[1401]R, has predicted MH2+ of 856.3561. In extracted ion intensity from the *Lrp2* p.C1401R MS1 spectra, there is a peptide with z=2 at m/z 856.3571 corresponding to the mutant peptide. **(B)** The tryptic peptide for *Lrp2* WT, TCEDVNECDIPGF[1401]CSQHCVNMR, has predicted MH2+ of 1364.5375. In extracted ion intensity from the *Lrp2* p.C1401R MS1 spectra, there is no evidence of a peptide with z=2 corresponding to the WT tryptic peptide. **(C)** MS2 spectra for *Lrp2* p.C1401R shows y-ion and b-ion ladders with clear peaks, confirming the identity of the mutant peptide. **(D)** The b-ion and y-ion ladders for the peptide are listed.

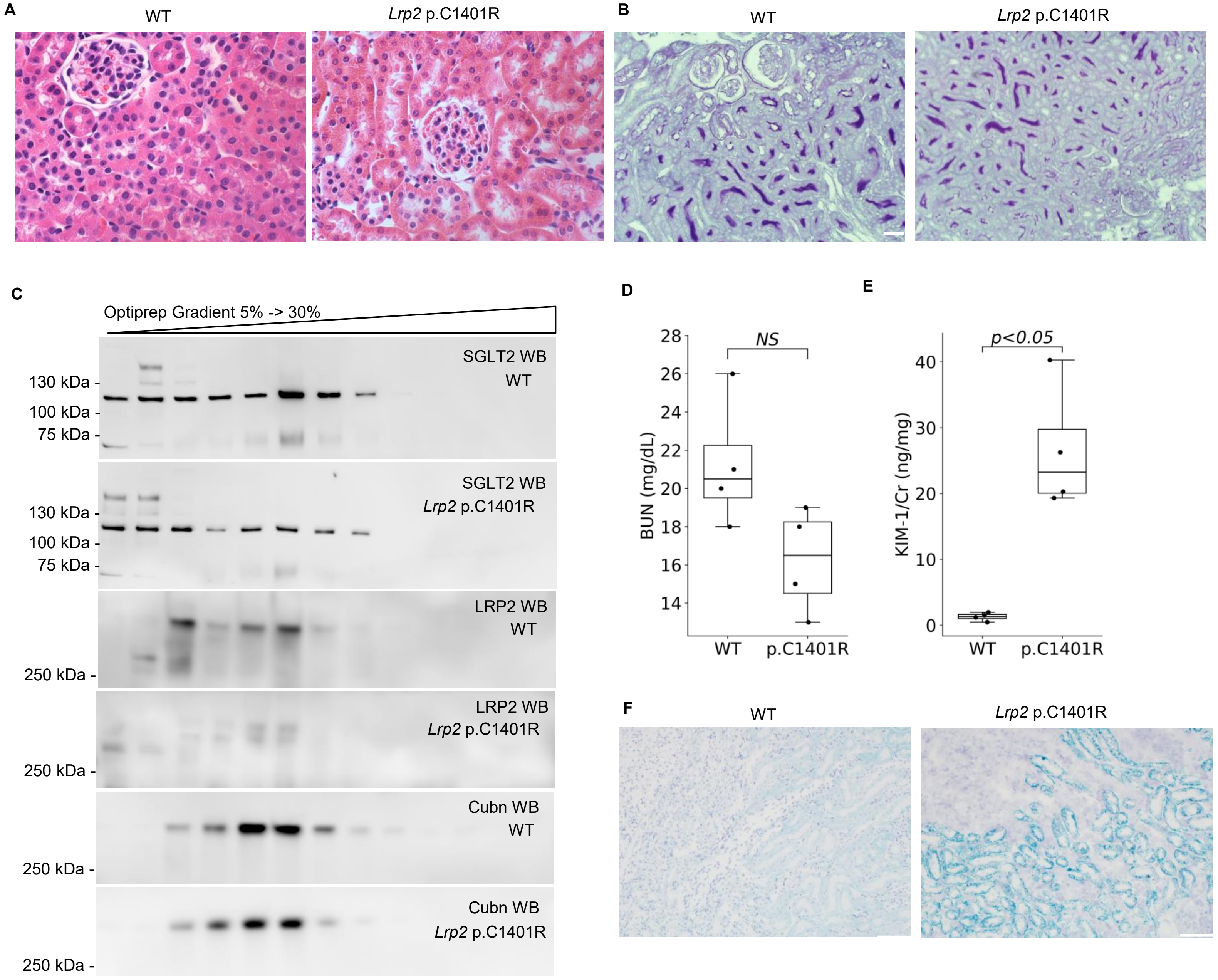


**Supplemental Figure 5: *Lrp2* p.C1401R shows evidence of tubular injury**

**(A)** Light microscopy of kidneys from WT and *Lrp2* p.C1401R mice with hematoxylin and eosin staining. Representative images, n=3 mice. Scale bar = 10 μm. **(B)** Light microscopy of kidneys from WT and *Lrp2* p.C1401R mice with PAS stain. Representative images, n=3 mice. Scale bar = 50 μm. **(C)** SGLT2, LRP2, and CUBN western blotting of WT and *Lrp2* p.C1401R PNS subjected to density gradient equilibrium ultracentrifugation in Optiprep medium with equal volume loading. **(D)** Box and whisker plot for BUN from WT (n=4) and *Lrp2* p.C1401R (n=4) mice, not significant using Welch's t-test. **(E)** Box and whisker plot for KIM-1 ELISA for urine from WT (n=4) and *Lrp2* p.C1401R (n=4) mice, p=0.013 using Welch's t-test. **(F)** RNAscope for *Havcr1* (KIM-1) on WT and *Lrp2* p.C1401R mice.

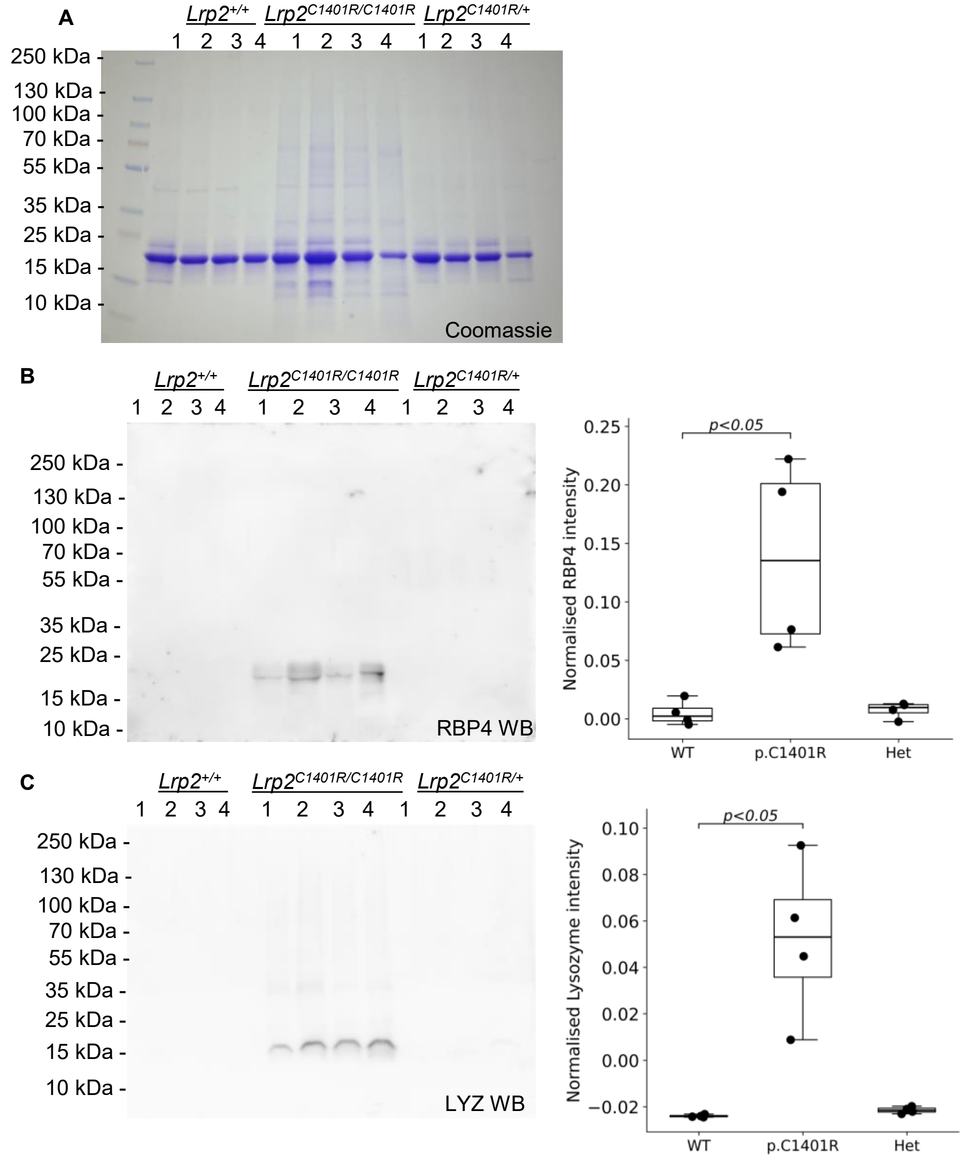


**Supplemental Figure 6: Proteinuria in *Lrp2* p.C1401R mice. (A)** Reducing, denaturing, SDS-PAGE 4-20% gradient gel with Coomassie blue staining showing increased low molecular weight (LMW) proteinuria in *Lrp2* p.C1401R mice compared to controls. n=4 mice each for WT, *Lrp2* p.C1401R homozygous (*Lrp2*$^{C1401R/C1401R}$), and *Lrp2* p.C1401R heterozygous mice (*Lrp2*$^{C1401R/+}$). For all panels, loading was controlled by urine creatinine. Quantitative western blotting of mouse urine for (**B**) Retinol-binding protein (RBP4) and (**C**) lysozyme (LYZ), showing significant increases in normalized intensity by Welch's t-test.

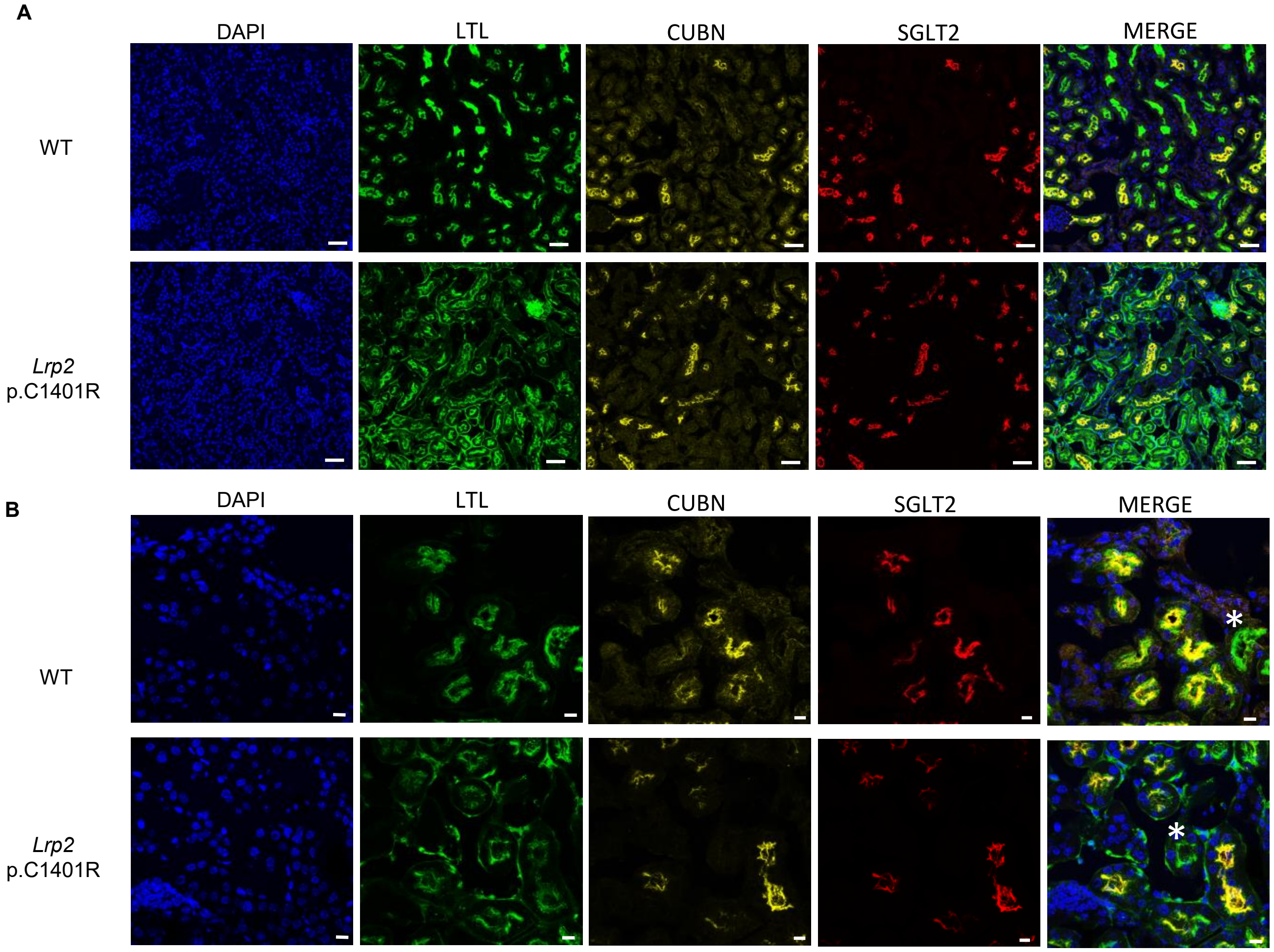


**Supplemental Figure 7: Cubilin localization preserved in *Lrp2* p.C1401R relative to WT**

**(A)** Immunofluorescence of WT and Lrp2 p.C1401R mice staining for DAPI, Lotus tetragonolobus lectin (LTL), cubilin (CUBN), and Sodium-glucose co-transporter 2 (SGLT2). Representative images from n=3 mice. Scale bar = 50 μm (**B**) High-magnification immunofluorescence from sections in panel A. SGLT2-/LTL+ S3 tubules are marked with white asterisks. Representative images from n=3 mice. Scale bar = 10 μm.

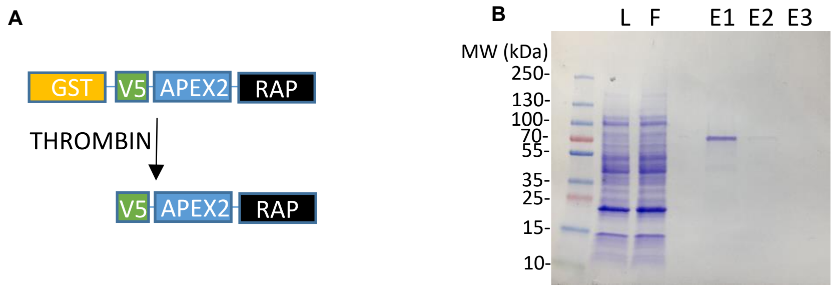


**Supplemental Figure 8: Production of V5-APEX2-RAP fusion protein. (A)** Schematic showing V5-APEX2-RAP protein construct before and after thrombin digestion. **(B)** Coomassie blue stained SDS-PAGE gel of lysate (L), Flow-through (F), and 3 eluates (E1, E2, E3) following GST capture and on-column thrombin digestion. Size markers (kDa) are at the left. The calculated size of V5-APEX2-RAP is 64 kDa.
