# Supplementary material for: Failure of endocytic flux in Donnai-Barrow syndrome caused by LRP2 p.C1400R
Source: JCI Insight. 2026 Apr 23;11(12):e199341. doi: 10.1172/jci.insight.199341 (PMC13313491; doi:10.1172/jci.insight.199341)
Supplement: Unedited blot and gel images [file jciinsight-11-199341-s097.pdf]

Figure 2B

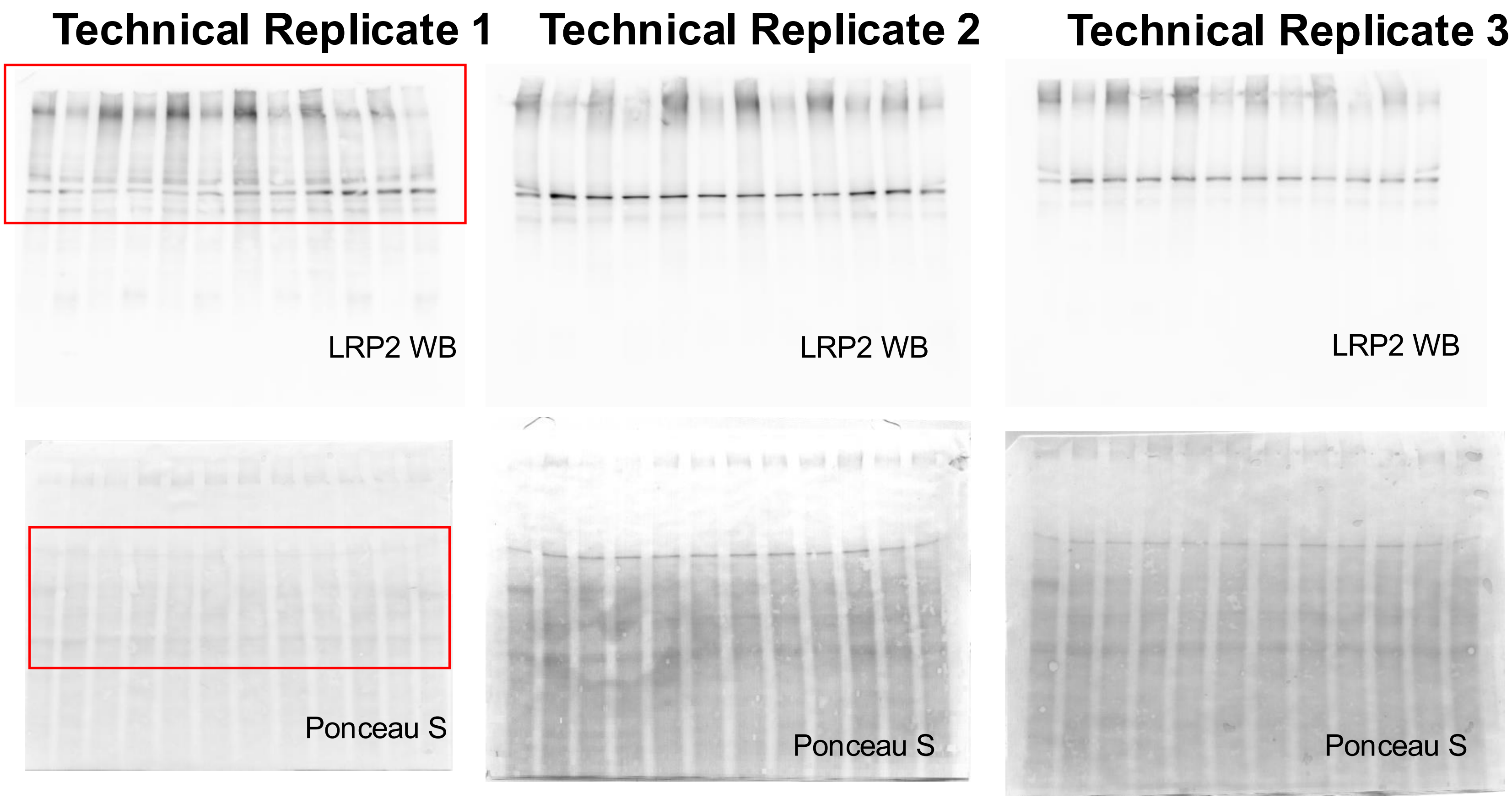
Technical Replicate 1
Technical Replicate 2
Technical Replicate 3
LRP2 WB
LRP2 WB
LRP2 WB
Ponceau S
Ponceau S
Ponceau S

Figure 3B

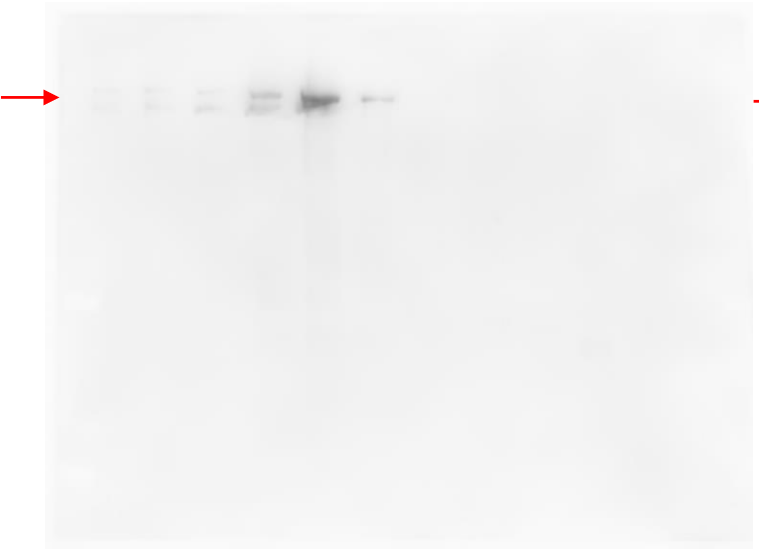

LRP2 WB

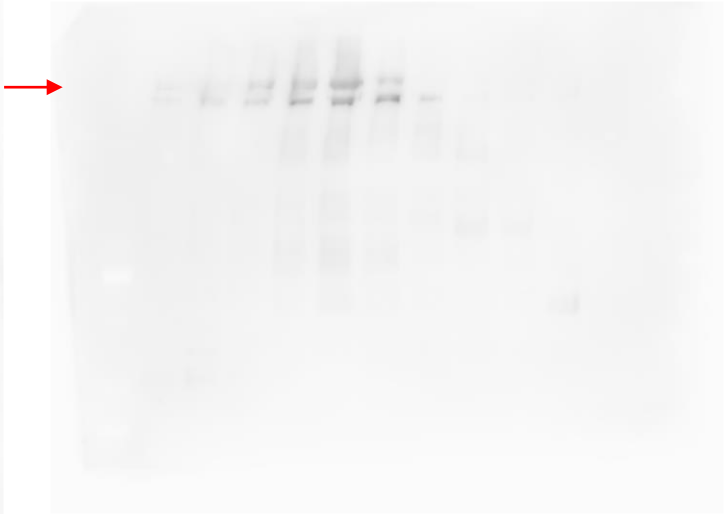

LRP2 WB

Supplementary Figure 5C

SGLT2 WB

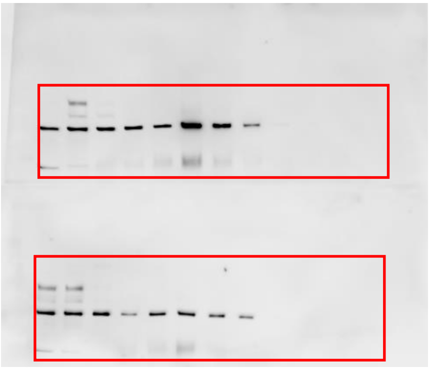

CUBN WB

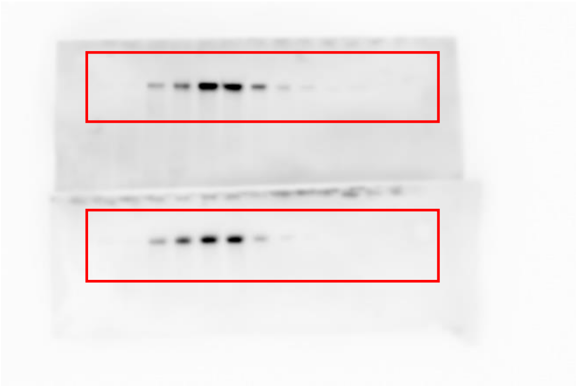

LRP2 WB

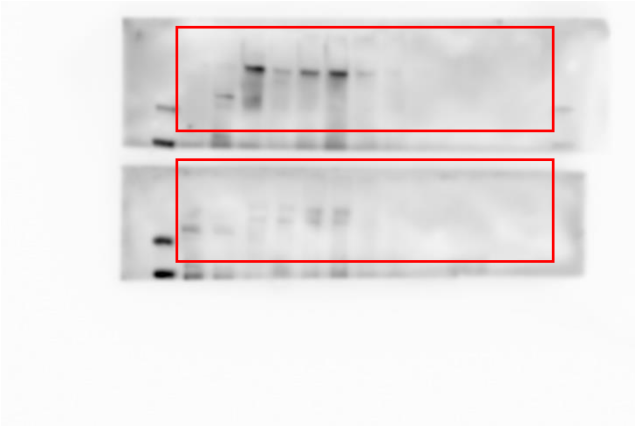

Supplementary Figure 6

S6A

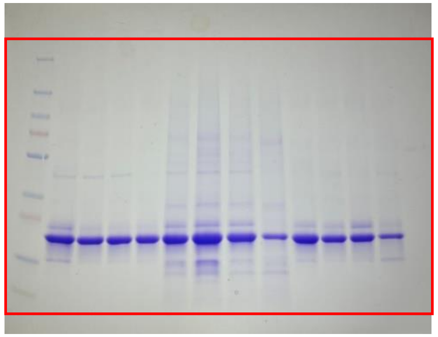

S6B

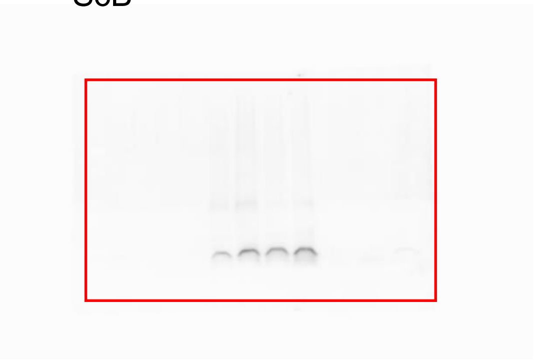

LYZ WB

S6C

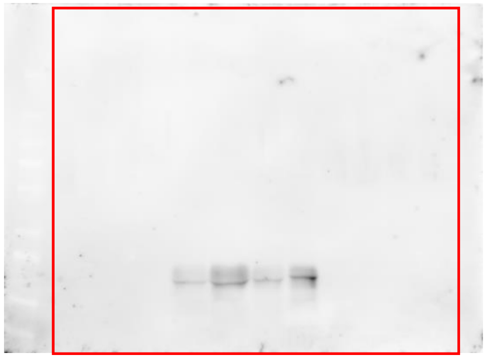

RBP4 WB
